# A meta analysis of genome-wide association studies for limb bone lengths in four pig populations

**DOI:** 10.1186/s12863-015-0257-1

**Published:** 2015-07-29

**Authors:** Yuanmei Guo, Lijuan Hou, Xufei Zhang, Min Huang, Huirong Mao, Hao Chen, Junwu Ma, Congying Chen, Huashui Ai, Jun Ren, Lusheng Huang

**Affiliations:** State Key Laboratory for Pig Genetic Improvement and Production Technology, Jiangxi Agricultural University, Nanchang, 330045 China; Current address: Wenzhou Medical University, WenZhou, 325000 China

**Keywords:** Limb bone length, Meta analysis, Pig, Genome-wide association study

## Abstract

**Background:**

Limb bone length is an economically important trait in pigs, because it is negatively correlated with backfat thickness, and is also a determinant to the yield of hip and loin. Moreover, abnormal growth of the limb bone leads to leg structural weakness. Until now, the genetic architecture of the pig lime bone length remains poorly understood. The object of this study was to map genomic loci for limb bone length by genome-wide association study (GWAS) on 4 pig populations.

**Results:**

We measured the lengths of five limb bones including scapula, humerus, ulna, femur and tibia that were dissected from the right-side carcass of 925, 331, 314 and 434 animals from White Duroc × Erhualian F_2_ intercross, Erhualian, Laiwu and Sutai populations, respectively. We genotyped the 2004 pigs for 62,163 single nucleotide polymorphisms (SNPs) on the Porcine SNP60 BeadChip, and performed GWAS and a GWAS meta analysis in the 4 populations. In total, we identified 12 and 4 loci associated with the limb bone lengths at suggestive and genome-wide significant levels respectively, of which 4 loci were reported for the first time. The most prominent locus was identified in a 924-kb (kilo base pairs) linkage disequilibrium block on *Sus Scrofa* chromosome (SSC) 7, and *High Mobility Group AT-hook 1* (*HMGA1*) appears to be a strong candidate gene in this region. Another promising locus is located in the middle of SSC4, and *Pleiomorphic Adenoma Gene 1* (*PLAG1*) is a functionally plausible candidate gene underlying the locus. Because the lengths of the 5 limb bones are highly correlated to each other, most of significant loci were associated with all of the 5 traits; however, several loci showed specific effect on the length of one limb bone, such as the locus at the proximal end of SSC2 associated with only the scapula length.

**Conclusion:**

To our knowledge, this study was the first GWAS meta analysis for limb bone lengths in pigs. As expected, the meta analysis is more powerful to identify genomic loci. A total of 16 loci were identified in this study, including four novel loci. *HMGA*1 and *PLAG*1 are two appearing candidate genes for pig limb bone lengths, which warrant further investigations.

**Electronic supplementary material:**

The online version of this article (doi:10.1186/s12863-015-0257-1) contains supplementary material, which is available to authorized users.

## Background

Limb bone length is a major factor influencing body height in pigs. It has been reported that body height is negatively correlated with backfat thickness, and is also a determinant to the yield of hip, loin, picnic shoulder and shoulder butt [1, 2]. Abnormal growth of the limb bone could lead to leg structural weakness, and unsound legs would cause huge economic loss, especially in breeding pigs [3].

In humans, body height is positively and highly correlated with limb bone length, with correlation coefficients from 0.66 to 0.88 [4, 5]. The heritability of human height is estimated to be above 0.8, but the top 697 associated SNPs collectively explain only 19.6 % of height heritability in human genome-wide association studies, known as the missing heritability [6, 7]. The domestic pig is an important large-animal model for studying complex traits in human as the pig shares many physiological similarities with human. Deciphering the genetic basis of limb bone growth in pigs will provide insight to understanding of the complex genetic architecture of human height.

Limb bone length is a multi-factorial trait in pigs. To date, a total of 39 quantitative trait loci (QTL) for limb bone length have been identified across the pig genome [8–10]. With the availability of Illumina PorcineSNP60 Beadchip [11], genome-wide association studies have been conducted on a variety of traits to improve the resolution of traditional QTL mapping [12]. GWAS meta analysis based on multiple populations can further increase the detection power and reduce false-positive findings by utilizing information from multiple independent studies [13]. To our knowledge, only one GWAS has been performed for pig limb bone length in an F_2_ population produced by a Large White × Minzhu intercross[10].

The aim of this study was to identify genomic loci for the lengths of limb bones including scapula, humerus, ulna, femur and tibia by GWAS and a meta GWAS analysis in four pig experimental populations: a White Duroc × Erhualian F_2_ intercross, Chinese Erhualian, Laiwu and Sutai pigs.

## Results

### Descriptive statistics of phenotypic traits

Descriptive statistics of phenotypic traits measured in the four pig populations are shown in Table 1. In general, the limb bones are longer (*P* < 0.05) in males than in females across those populations. An additional table shows the phenotypic correlation coefficients among the 5 measured traits related to limb bone length [see Additional file 1]. All of the phenotypic correlation coefficients were positive and highly significant (*P* < 0.0001).

**Table 1:** Descriptive statistics and the differences between sexes of the limb bone lengths

	Male		Female		Male - Female		All
	*n*	Mean ± S.E.	*n*	Mean ± S.E.	Mean ± S.E.	*P* value	Mean ± S.E.
Scapula length, cm							
Erhualian	166	20.89 ± 0.114	165	20.95 ± 0.098	−0.059 ± 0.1501	0.6927	20.92 ± 0.075
F_2_	510	22.60 ± 0.067	415	22.17 ± 0.080	0.425 ± 0.1032	4.8 × 10^−5^	22.41 ± 0.052
LaiWu	216	21.27 ± 0.073	98	20.63 ± 0.115	0.633 ± 0.1334	6.8 × 10^−6^	21.07 ± 0.064
SuTai	225	19.68 ± 0.081	201	19.34 ± 0.081	0.340 ± 0.1143	0.0030	19.52 ± 0.058
Humerus length, cm							
Erhualian	166	17.81 ± 0.091	165	17.70 ± 0.072	0.108 ± 0.1160	0.3530	17.75 ± 0.058
F_2_	510	18.71 ± 0.055	414	17.94 ± 0.058	0.767 ± 0.0801	5.5 × 10^−21^	18.36 ± 0.042
LaiWu	216	17.93 ± 0.060	98	17.18 ± 0.104	0.748 ± 0.1134	4.1 × 10^−9^	17.70 ± 0.056
SuTai	228	17.24 ± 0.068	206	16.82 ± 0.070	0.427 ± 0.0974	1.4 × 10^−5^	17.04 ± 0.050
Ulna length, cm							
Erhualian	166	19.13 ± 0.098	165	18.82 ± 0.085	0.308 ± 0.1294	0.0177	18.97 ± 0.065
F_2_	510	20.45 ± 0.065	415	19.65 ± 0.072	0.808 ± 0.0968	2.7 × 10^−16^	20.09 ± 0.050
LaiWu	216	19.08 ± 0.066	98	18.36 ± 0.105	0.722 ± 0.1204	2.6 × 10^−8^	18.85 ± 0.059
SuTai	228	18.60 ± 0.073	206	18.09 ± 0.074	0.504 ± 0.1039	1.7 × 10^−6^	18.36 ± 0.053
Femur length, cm							
Erhualian	166	20.46 ± 0.124	165	20.33 ± 0.081	0.126 ± 0.1479	0.3956	20.40 ± 0.074
F_2_	510	21.14 ± 0.057	414	20.72 ± 0.066	0.421 ± 0.0871	1.8 × 10^−6^	20.95 ± 0.044
LaiWu	216	20.59 ± 0.065	98	19.91 ± 0.102	0.686 ± 0.1190	6.4 × 10^−8^	20.38 ± 0.058
SuTai	228	19.68 ± 0.075	206	19.33 ± 0.074	0.354 ± 0.1053	0.0008	19.51 ± 0.053
Tibia length, cm							
Erhualian	166	18.34 ± 0.089	165	18.05 ± 0.075	0.291 ± 0.1169	0.0134	18.20 ± 0.059
F_2_	509	18.97 ± 0.053	413	18.66 ± 0.061	0.314 ± 0.0808	0.0001	18.83 ± 0.041
LaiWu	216	18.58 ± 0.059	98	17.77 ± 0.090	0.807 ± 0.1064	2.6 × 10^−12^	18.33 ± 0.054
SuTai	228	18.02 ± 0.070	206	17.57 ± 0.075	0.448 ± 0.1023	1.5 × 10^−5^	17.81 ± 0.052

### Distribution of the Z value in the meta analysis

In this study, we used a *Z* test to detect the association between SNPs and limb bone lengths in the GWAS meta analysis. The Kolmogorov-Smirnov test results showed that all of the statistics followed the standard normal distribution except the one of the ulna length (*P* = 0.0448) [see Additional file 2].

### SNP quality control results

After quality control, 39461, 28094, 36585 and 41320 SNPs were retained for GWAS on the F_2_ (*n* = 925), Erhualian (*n* = 331), Laiwu (*n* = 314) and Sutai (*n* = 434) populations, respectively [see Additional file 3]. The average physical distances between adjacent SNPs were 74.7, 105.1, 80.7 and 71.2 kb in the four populations, respectively. In the meta GWAS analysis, a common set of 15429 SNPs in the four populations was used, with an average physical distance between adjacent SNPs was 189.9 kb.

### Principal component analysis results

The principal component analysis showed that no population stratification existed in the F_2_ population, but the other populations had a certain level of population stratifications, especially in Erhualian and Sutai pigs [see Additional file 4]. To remove the effect of population stratifications, a random polygenic effect estimated with a genomic kinship was included in the GWAS model, and the residual inflation was corrected by genomic control. The effect of the population stratification was completely corrected in the four populations [see Additional file 5].

### Genetic distances among individuals

The individuals were clustered into four groups according to their genetic distances [see Additional file 6], and the clustering results completely agreed with the populations without exception. The Erhualian and the Sutai were clustered into a group and the rest two populations into another group.

### Single-population GWAS results

**Fig. 1:**
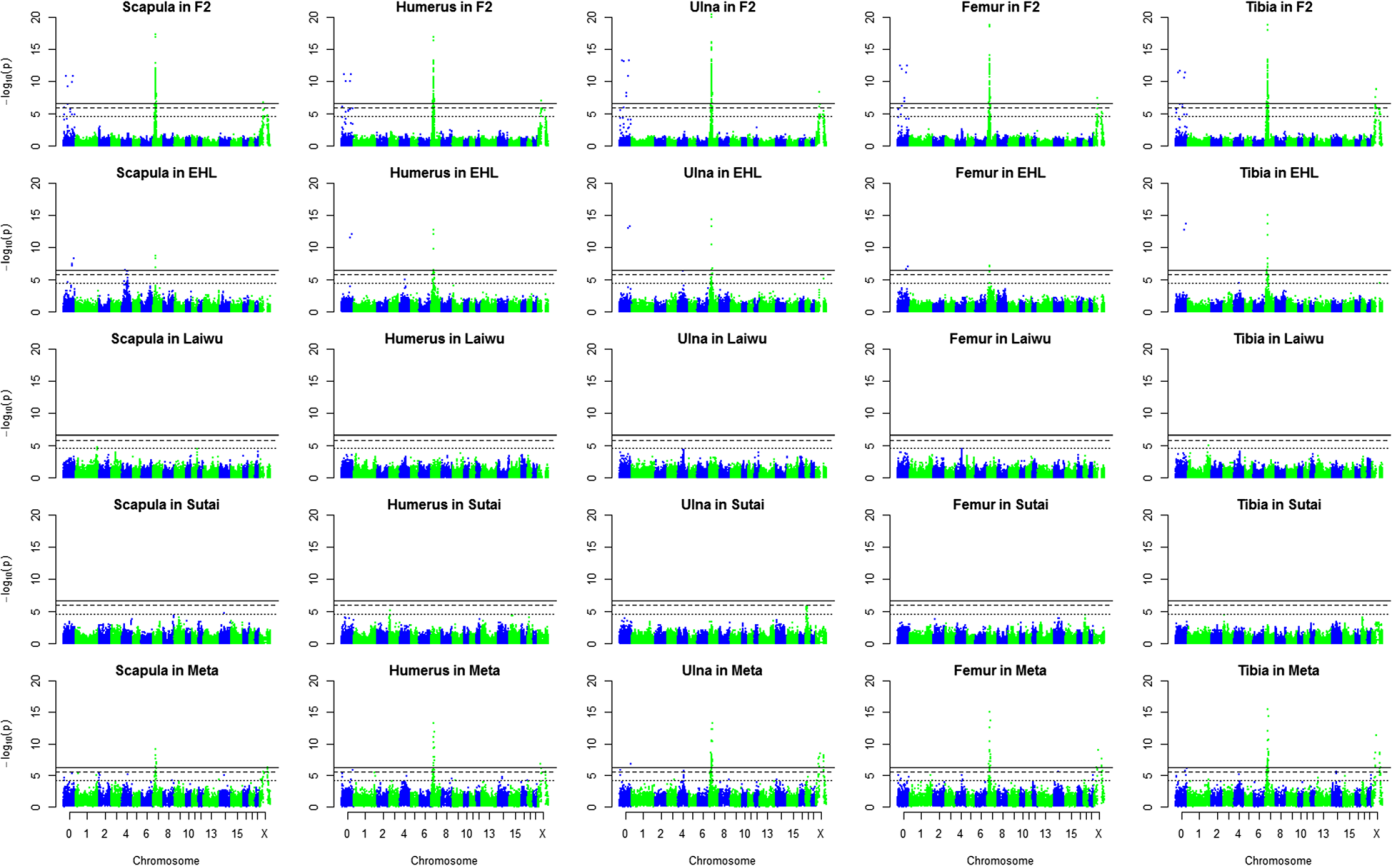
Manhattan plots of GWAS for limb bone lengths. The solid, dashed and dotted horizontal lines indicate the 1 % and 5 % genome-wide and chromosome-wise (suggestive) significant threshold values, respectively

In single-population analyses, we identified 3 and 4 chromosomal regions associated with limb bone lengths at suggestive and genome-wide significant levels, respectively (Table 2, Fig. 1). In the F_2_ we detected two loci associated with all of the 5 traits at 1 % genome-wide significant level. One was around 35 mega base pairs (Mb) on SSC7, and the other was in the middle of X chromosome. For the analysis in the Erhualian, we detected three QTL. One for all of the 5 traits was mapped around 35 Mb on SSC7. Another one for the three front-limb bone lengths was detected around 66 Mb on SSC4. The third one for the ulna length was identified around 103 Mb on the X chromosome. For the Laiwu, only one locus at the distal end of SSC1 was significantly associated with the lengths of scapula and tibia. Another region around 77 Mb on SSC4 had an association with the lengths of femur and ulna almost reaching the suggestive significant level (Fig. 1). For the Sutai, we identified three chromosomal regions affecting limb bone lengths. One for the humerus length was mapped around 9 Mb on SSC3. Another locus around 65 Mb on SSC14 was associated with the scapula length. The third one located around 14 Mb on SSC17 and had a significant effect on ulna length. To our knowledge, there is no QTL reported for limb bone length on this chromosome.

**Table 2:** The chromosomal regions significantly associated with the limb bones lengths

Chr^1^	Trait	Population	Top SNP	Position, Mb	Effect ± S.E.	*P* Value^2^	*N*_SNP_^3^
1	Scapula	Meta	ss131151852	30.72	0.186 ± 0.041	5.43 × 10^−6^	1
1	Scapula	Meta	ss131149408	282.04	0.171 ± 0.039	1.10 × 10^−5^	1
1	Tibia	Meta	ss131148700	279.60	0.136 ± 0.033	4.38 × 10^−5^	1
1	Tibia	Laiwu	ss107826847	280.64	0.286 ± 0.059	1.12 × 10^−5^	1
1	Femur	Meta	ss131151073	285.76	0.150 ± 0.034	9.68 × 10^−6^	1
1	Humerus	Meta	ss131151073	285.76	0.144 ± 0.031	4.11 × 10^−6^	2
1	Scapula	Laiwu	ss131102194	299.48	0.300 ± 0.069	2.15 × 10^−5^	1
2	Scapula	Meta	ss131184380	4.87	0.144 ± 0.032	6.06 × 10^−6^	3
3	Humerus	Sutai	ss131215509	9.48	0.344 ± 0.075	7.14 × 10^−6^	1
3	Ulna	Meta	ss131222600	97.95	0.138 ± 0.034	4.99 × 10^−5^	1
4	Scapula	Erhualian	ss131260763	42.51	0.965 ± 0.173	2.87 × 10^-7**^	10
4	Tibia	Meta	ss478940901	63.32	0.162 ± 0.036	7.61 × 10^−6^	1
4	Humerus	Erhualian	ss478940911	65.72	0.478 ± 0.104	1.18 × 10^−5^	1
4	Ulna	Erhualian	ss478940911	65.72	0.648 ± 0.116	4.37 × 10^-7*^	1
4	Ulna	Meta	ss131269439	81.71	0.195 ± 0.041	1.93 × 10^-6*^	6
4	Femur	Meta	ss131269533	81.94	0.173 ± 0.039	8.61 × 10^−6^	2
4	Scapula	Meta	ss478941011	82.53	0.187 ± 0.042	7.44 × 10^−6^	1
4	Humerus	Meta	ss131270306	84.98	0.164 ± 0.038	1.41 × 10^−5^	1
7	Femur	Meta	ss131342998	33.79	0.316 ± 0.039	9.99 × 10^-16**^	32
7	Humerus	Meta	ss131342998	33.79	0.261 ± 0.035	5.53 × 10^-14**^	35
7	Scapula	Meta	ss131342998	33.79	0.255 ± 0.041	6.67 × 10^-10**^	18
7	Tibia	Meta	ss131342998	33.79	0.317 ± 0.036	5.53 × 10^-14**^	44
7	Ulna	Meta	ss131342998	33.79	0.347 ± 0.039	5.53 × 10^-14**^	48
7	Femur	F_2_	ss107837325	34.80	0.884 ± 0.075	1.71 × 10^-19**^	109
7	Scapula	F_2_	ss107837325	34.80	1.029 ± 0.091	4.85 × 10^-18**^	110
7	Femur	Erhualian	ss131343870	34.84	0.557 ± 0.096	7.05 × 10^-8**^	3
7	Humerus	Erhualian	ss131343870	34.84	0.553 ± 0.071	1.84 × 10^-13**^	19
7	Scapula	Erhualian	ss131343870	34.84	0.605 ± 0.093	2.04 × 10^-9**^	3
7	Tibia	Erhualian	ss131343870	34.84	0.644 ± 0.073	1.05 × 10^-15**^	28
7	Ulna	Erhualian	ss131343870	34.84	0.679 ± 0.078	4.51 × 10^-15**^	17
7	Humerus	F_2_	ss107806758	35.18	0.818 ± 0.074	1.39 × 10^-17**^	102
7	Tibia	F_2_	ss107806758	35.18	0.872 ± 0.073	1.68 × 10^-19**^	109
7	Ulna	F_2_	ss107806758	35.18	1.129 ± 0.086	3.73 × 10^-21**^	108
8	Ulna	Meta	ss107904229	133.84	0.148 ± 0.036	4.23 × 10^−5^	1
8	Humerus	Meta	ss131065181	145.99	0.131 ± 0.032	3.86 × 10^−5^	1
13	Tibia	Meta	ss131486660	29.08	0.156 ± 0.037	2.58 × 10^−5^	1
13	Scapula	Meta	ss131483019	214.15	0.151 ± 0.038	5.62 × 10^−5^	1
14	Ulna	Meta	ss131520568	10.50	0.131 ± 0.032	4.79 × 10^−5^	1
14	Femur	Meta	ss131515687	64.87	0.179 ± 0.038	2.46 × 10^-6*^	1
14	Scapula	Meta	ss131515687	64.87	0.178 ± 0.040	8.46 × 10^−6^	1
14	Scapula	Sutai	ss131515687	64.87	0.261 ± 0.059	2.00 × 10^−5^	1
14	Humerus	Meta	ss131516260	67.34	0.137 ± 0.032	1.90 × 10^−5^	2
14	Tibia	Meta	ss131516260	67.34	0.150 ± 0.032	2.66 × 10^-6*^	3
15	Tibia	Meta	ss478943357	20.82	0.127 ± 0.029	1.45 × 10^−5^	1
17	Femur	Meta	ss131554491	10.79	0.126 ± 0.032	6.26 × 10^−5^	1
17	Ulna	Sutai	ss131542878	13.79	0.508 ± 0.103	1.97 × 10^−6^	8
17	Ulna	Meta	ss131546319	37.50	0.192 ± 0.045	1.93 × 10^−5^	1
18	Femur	Meta	ss107835048	52.79	0.147 ± 0.033	9.83 × 10^−6^	2
18	Tibia	Meta	ss107835048	52.79	0.135 ± 0.031	9.52 × 10^−6^	2
X	Humerus	Meta	ss478936157	41.48	0.113 ± 0.021	1.47 × 10^-7**^	8
X	Femur	F_2_	ss478944418	43.40	0.198 ± 0.028	3.73 × 10^-8**^	9
X	Humerus	F_2_	ss478944418	43.40	0.191 ± 0.027	9.02 × 10^-8**^	16
X	Scapula	F_2_	ss478944418	43.40	0.229 ± 0.034	1.81 × 10^-7**^	9
X	Ulna	F_2_	ss478944418	43.40	0.241 ± 0.030	4.85 × 10^-9**^	16
X	Femur	Meta	ss23131102	63.65	0.133 ± 0.022	8.67 × 10^-10**^	15
X	Tibia	Meta	ss23131102	63.65	0.140 ± 0.020	4.67 × 10^-12**^	20
X	Ulna	Meta	ss23131102	63.65	0.129 ± 0.022	3.55 × 10^-9**^	25
X	Tibia	F_2_	ss23131102	63.65	0.249 ± 0.031	1.66 × 10^-9**^	24
X	Ulna	Erhualian	ss478935724	102.94	0.223 ± 0.045	7.08 × 10^−6^	1
X	Scapula	Meta	ss131570200	107.48	0.114 ± 0.023	6.21 × 10^-7**^	15

Notes: 1. Chromosome; 2. The *P* value was corrected by genomic control. **: 1 % genome-wide significant; *: 5 % genome-wide significant; without *: suggestive significant; 3. number of SNPs that surpass the suggestive significance level.

### Comparing single-population GWAS results

We didn’t identify any significant locus common across all of the four tested populations, but two significant loci each on SSC7 and X were shared by the F_2_ and Erhualian populations (Table 2, Fig. 1). This may be due to the fact that Erhualian is one of the two founder breeds of the F_2_ population. Another locus around 77 Mb on SSC4 was shared by the Erhualian, F_2_ and Laiwu populations (Table 2, Fig. 1 and Additional file 7). Although this locus was not significant in the Laiwu population, its signal was clear and its lead SNP almost reached the suggestive significant level. This locus was also uncovered in the F_2_, because it was concealed by the noises from the effects of the major loci on SSC7 and X. After removed those noises, the locus was finally detected.

### GWAS meta-analysis results

Under the assumption of the linkage phases being different across populations, we identified a total of 15 chromosomal regions significantly associated with the measured traits. Those loci replicated all of the loci identified by the single-population analysis except the locus associated with the ulna length at the proximal end of SSC3. Four loci were found to be associated with limb bone lengths for the first time, which were the locus at the distal end of SSC8 for the lengths of humerus and ulna, the locus at the distal end of SSC13 for scapula length, the locus at the proximal end of SSC17 and the locus in the middle of SSC18 for the lengths of femur and tibia. We also performed a meta analysis across the 3 pure breeds, and the results were similar to those across 4 populations except that the loci’s significant levels were slightly low [see Additional file 8].

Under the assumption of the linkage phases being same across populations, 8 loci were detected including the three loci on SSC8, 17 and 18 reported for the first time [see Additional file 9], and all of them had been identified under the other assumption.

### Chromosomal regions with P < 0.001

Since Bonferroni-corrected thresholds are very conservative, using the Bonferroni correction will decrease the detection power. To overcome it, some loci with significant levels slightly lower (*P* < 0.001, corrected by genomic control) than the suggestive level were listed in an additional table [see Additional file 10].

### GWAS results after fixed the lead SNPs on SSC7 and X in the F_2_ population

When we included the lead SNPs on SSC7 and X as fixed effects in the GWAS model, we identified an additional chromosome-wise significant locus at 84.2 Mb on SSC4 and some weak signals on other chromosomes, such as SSC1 and 8 [see Additional file 7].

### Linkage disequilibrium block on SSC7

The top SNP located in a 1941-kb linkage disequilibrium (LD) block in the F_2_ and in a 924-kb LD block in the Erhualian [see Additional file 11]. The LD block of the F_2_ completely encompassed the LD block of the Erhualian, so the locus was fine mapping in a 924 kb interval. Although the four populations shared a 93-kb LD block from 34556148 to 35814316 base pairs, we didn’t guarantee the interval containing the causative gene because there was no signal detected in the other two populations.

### Two-point linkage analysis results

There were 20 unmapped SNPs associated with limb bone lengths at the suggestive significant level [see Additional file 12]. Those SNPs consolidated the associating signals on SSC7 in the F_2_, on SSC4 and 7 in the Erhualian, and on SSC1, 4 and 7 in the meta analysis. The unmapped SNP ss107830715, which tightly links to the SNP ss107865741 at 15.10 Mb on SSC17, added an associating signal to the tibia length on SSC17 in the meta analysis.

## Discussion

In this study, we conducted GWAS and GWAS meta analyses in four pig experimental populations. In total, we identified 16 loci significantly associated with the limb bone lengths, of which 12 confirmed the QTL previously reported on SSC1, 2, 3, 4, 7, 13, 14, 15, and X [8–10], and four loci were reported for the first time.

### QTL results vs GWAS results in the F_2_ population

Our previous QTL mapping study detected 35 QTL for the five traits on 11 chromosomes in the F_2_ population [9]. In this study, we detected only 2 chromosomes associated with those traits in the same F_2_ population. The lower power of GWAS could be explained by the following factors. First, the Bonferroni-corrected thresholds in GWAS are more conservative than those obtained from the permutation test used in QTL mapping studies. Second, the error variance is smaller in the QTL mapping study than in the GWAS. When perform genome scan for QTL, the detected QTL in the previous rounds are included in the model as fixed effects, so their variances are removed from the error variance [14]. However, these kinds of variances are not removed from the error variance in GWAS. Third, the statistic model for QTL mapping fixed the additive, dominant and imprinting effects of a locus, while the GWAS model included only the additive effect.

When we used a looser stringent threshold (*P* < 0.001, corrected by genomic control), we didn’t detected more locus in the F_2_ [see Additional file 10]. But when we corrected the effects of the two major loci by including their lead SNPs as fixed effects in the GWAS model, we identified an additional chromosome-wise significant locus on SSC4 and some other weak signals on other chromosomes [see Additional file 7]. This indicates that the significant effects of the loci on SSC7 and SSCX ruined the signals on other chromosomes. Therefore, if a trait is affected by few major loci, corrections of effects of these loci are required to improve the detection power of GWAS.

### Single-population GWAS vs GWAS meta analysis

To our knowledge, this is the first GWAS meta analysis of limb bone lengths in pigs. We used a common set of informative SNPs in the four populations to conduct the GWAS meta analysis. Although the SNP density in the GWAS meta analysis is lower than that used in a single population, the meta analysis is more powerful to identify chromosomal regions associated with limb bone lengths than the single-population GWAS. In the single-population GWAS, the detection power is limited by the small population size (In spite there are more than 900 F_2_ individuals, the major loci on SSC7 and X ruin the signal on other chromosomes), and the locus with small effect can be unveiled by single-population analysis. The meta analysis utilizes information from multiple independent studies and can increase the detection power. It not only detected 8 more regions than the single population analyses, but also confirmed all loci identified in the four populations separately except the locus at the proximal end of SSC3.

### Linkage phases being same vs different across populations

Considering most of SNPs on the porcine 60 K SNP Beadchip are not the causative mutation for the five traits, so it is more reasonable to assume the linkage phases between the SNP and the causative mutation being different than being same across the four populations. Under the “different” assumption, we identified 15 loci for the five traits. While under the “same” assumption, just 8 of the 15 loci were detected and no other locus was identified. Therefore, the former assumption can significantly improve the power.

### Comparing the results among traits

As the five traits related to limb bone lengths are highly correlated [see Additional file 1], it is expected to identify chromosomal regions associated with all these traits, such as the region around 35 Mb on SSC7 in the F_2_ and Erhualian populations (Table 2). However, some chromosomal regions are associated with one of the five traits. For instance, the region at the proximal end of SSC2 is only associated with the scapula length. This indicates that both common and specific genes are involved in the growths of different limb bones.

### Candidate genes underlying promising loci

The locus around 35 Mb on SSC7 showed the most significant effect on the length of all limb bones. This locus has a pleiotropic effect on multiple traits, including the gait scores of front and rear legs [15], fat acid compositions [16], skin thickness [17], feed efficiency [18], growth and fatness [19]. The top SNPs locate in a 924-kb LD block in the F_2_ and Erhualian populations [see Additional file 11], and 14 genes locate in this region. Recently, the confidence interval of this multi-faced locus has been refined into a 750-kb region that harbors the high mobility group AT-hook 1 gene (*HMGA*1) [19], which influences human’s height through chromatin structure modification [20]. Another member of high mobility group AT-hook family *HMGA*2 was also reported associated with human’s height [21]. Therefore, *HMGA*1 is a strong candidate gene for limb bone lengths on SSC7.

On chromosome X, the top associated SNPs of the five traits covered a 64.1-Mb region (from 43.4 to 107.5 Mb). It was probably caused by the extremely low recombination rate and low SNP density in this region. This region encompasses a big recombinant cold spot [22], in which SNPs are in highly linkage disequilibrium with each other. Furthermore, the lack of recombination usually results in many discarded SNPs that violate the Hardy-Weinberg equilibrium. The exceptionally large interval makes it impossible to highlight functionally plausible genes for the SSCX locus.

A region around 82 Mb on SSC4 was detected to be associated with the limb bone lengths in the GWAS meta analysis (Table 2) as well as in the F_2_ population [see Additional file 7]. Pleiomorphic adenoma gene 1 (*PLAG*1) locates about 600 kb away from the top SNP and has been reported to be associated with pig body length [23], bovine stature [24] and human height [25], so it is a strong candidate gene for this locus.

## Conclusions

In this study, we performed the first GWAS meta analysis for limb bone lengths in pigs. As expected, the meta analysis is more powerful to identify genomic loci than the single population GWAS. A total of 16 loci were identified in this study, including four novel loci. *HMGA*1 and *PLAG*1 are highlighted as novel candidate genes for pig limb bone lengths, and they are worthy of further investigations.

## Methods

### Ethics Statement

All procedures involving in the experimental animal are in compliance with guidelines for the care and use of experimental animals established by the Ministry of Agriculture of China. The ethics committee of Jiangxi Agricultural University specifically approved this study.

### Animals and Phenotypic Measurements

A total of 2004 pigs were used in this study including 925, 434, 331 and 314 animals from F_2_, Sutai, Erhualian and Laiwu populations respectively. The F_2_ and Sutai populations had been described in our previous publications [15, 26]. In brief, the F_2_ population was derived from a cross between two White Duroc boars and 17 Erhualian sows [26]. The Sutai pig is a Chinese synthetic breed that is originated from a cross between Duroc boars and Taihu sows, and has been selected for prolificacy and growth more than 18 generations [15]. Erhualian pigs were purchased from Jiaoxi Erhualian Specialized Cooperative Society in Jiangsu province in 2012. To cover most lineages of the Erhualian breed, 331 Erhualian individuals were selected from 11 sire and 53 dam families. Laiwu pigs (*n* = 314) were bought from Laiwu Stock Seed Farm in Shandong province from 2012 to 2013. Those pigs were derived from 11 sire and 45 dam families and covered almost all lineages of the Laiwu breed. Both Erhualian and Laiwu are Chinese indigenous breeds, the former is famous for its high prolificacy, and the latter is well known for its high intramuscular fat content. Erhualian and Laiwu boars were castrated before weaning, and females were intact. The Erhualian and Laiwu pigs at the age of 60 ± 3 days were transported from their original farms to Nanchang Guohong Ecological Farm in Jiangxi province. During the fattening period, both Erhualian and Laiwu pigs accessed *ad libitum* to the fresh water and a consistent feed, which met the pig’s needs for energies, amino acids, vitamins and minerals. Both Erhualian and Laiwu pigs were harvested at the age of 300 ± 3 days.

Scapula, humerus, ulna, femur and tibia were dissected from the right side carcass after the animal sacrificed at 240 ± 3 days in F_2_ and Sutai populations and at 300 ± 3 days in the other two populations. The lengths of these limb bones were measured according to the method described in Mao *et al.* [9]. Briefly, scapula length is the maximum straight line distance from the cavitas glenoidalis to the border of scapular cartilage. Humerus length is the length from the head to the trochlea. Ulna length is the distance from the olecranon process to the styloid process. Femur length is the length from the greater trochanter to the intercondyloid fossa. Tibia length is the distance from the intercondylar eminence to the medial malleolus.

### Genotyping and Quality Control

Genomic deoxyribonucleic acid (DNA) was extracted from ear or tail tissues under a standard phenol/chloroform protocol. All DNA samples were qualified and diluted to a concentration of 50 ng/μl. All of the animals from the four populations were genotyped by the porcine 60 K SNP Beadchip on an iScan System (Illumina, USA) following the manufacturer’s protocol. The SNP positions were determined according to the pig genome assembly Sscrofa10.2. The unmapped SNPs were assigned to chromosome 0, and their positions were arbitrarily given by the rule described in the reference [15]. SNPs were excluded if their call rate < 95 %, minor allele frequency < 5 %, or severely deviating from Hardy-Weinberg equilibrium (*P* < 0.000001), and the animals with call rate <95 % were removed. A common set of SNPs that passed quality control across the four populations were used in the GWAS meta analysis.

### Statistical methods

Descriptive statistics of the measured traits were calculated by the MEANS procedure. Phenotypic difference between sexes was tested by the TTEST procedure of SAS9.0 (SAS Institute Inc., USA). The CORR procedure was used to compute phenotypic correlation coefficients, and the MIXED procedure was engaged to determine the fixed effects and the covariates for the GWAS model. Sex and batch were included as fixed effects, and body weight at harvest was included as a covariate in the GWAS model for all traits. To remove the effect of the population stratification, a random polygenic effect estimated with a genomic kinship was included in the GWAS model [27, 28]. The genomic kinship was calculated based on the identity-by-state of the SNPs on autosomes [29, 30]. The genomic kinship was also used to estimate the genetic distances among individuals, and a phylogenetic tree was constructed by the heatmap function of the R program.

The GenABEL in the R package was used to conduct individual population GWAS using an additive model [31]. The family-based score test for association was used to detect the association between SNPs and traits, and the residual inflation was corrected by genomic control [32–34]. By applying Bonferroni correction, the *P* values for suggestive, 5 % and 1 % genome-wide significant levels were one, 0.05 and 0.01 divided by the number of informative SNPs, respectively.

The inverse variance pooling method was applied to perform a GWAS meta analysis of the four populations [35]. The weight (*w*_*i*_) for the *i*th population is calculated by the following formula:

$$ {w}_i=\frac{1}{s_i^2} $$

where *s*_*i*_ is the standard error of the allele effect in the *i*th population.

Then, the pooled estimate of the effect (*β*) and its standard error (*s*^2^) are calculated by the following formula:

$$ \begin{array}{l}\beta =\frac{{\displaystyle {\sum}_{i=1}^4{w}_i{\beta}_i}}{{\displaystyle {\sum}_{i=1}^4{w}_i}}\\ {}{s}^2=\frac{1}{{\displaystyle {\sum}_{i=1}^4{w}_i}}\end{array} $$

where *β*_*i*_ is the allele effect in the *i*th population.

A statistic of *Z*-test was calculated by the below formula under the assumption of the linkage phases being same across populations, and a Kolmogorov-Smirnov test was used to determine whether the statistic follows the standard normal distribution.

$$ Z=\frac{\beta }{s}=\frac{{\displaystyle {\sum}_{i=1}^4{w}_i{\beta}_i}}{\sqrt{{\displaystyle {\sum}_{i=1}^4{w}_i}}} $$

We used *β*_*i*_ and its absolute value to calculate the pooling *β* and *Z* values under the assumptions of the linkage phases being same and different across the populations, respectively. A Bonferroni correction was used to obtain the *P* values for suggestive, 5 % and 1 % genome-wide significant levels in the meta GWAS analysis.

The linkage between the mapped and unmapped associative SNPs was determined by two-point linkage analysis using CRI-MAP in the F_2_ population [36]. Two SNPs were defined as closely linked markers if the logarithm of the odd score was equal to or greater than 3. Haplotypes of target regions were inferred by Simwalk2.9 [37], and linkage disequilibrium blocks were defined using Haploview4.2 [38].

## Additional files

Additional file 1:
**The phenotypic correlation coefficients between the lengths of limb bones.** This table provides the phenotypic correlation coefficients between the limb bone lengths in the four tested pig populations. (PDF 61 kb) Additional file 2:
**The distribution of the statistic (Z value) using in the GWAS meta analysis.** This figure shows the distribution of *Z* value using in the meta-analysis. All of the statistics approximately follow the standard normal distribution. (PDF 144 kb) Additional file 3:
**The numbers of animals and SNPs passed through the quality control.** This table lists the SNP and animal numbers and the average physical distance interval of adjacent SNPs using in the GWAS analysis. (PDF 113 kb) Additional file 4:
**The schematic diagram of the first two principle components based on the genomic kinships.** This figure presents the population stratification based on the genomic kinships in the four populations. (PDF 65 kb) Additional file 5:
**The quantile-quantile plot for the limb bone lengths in the four populations.** This figure indicates the residual inflation has been completely corrected by genomic control in GWAS. (PDF 265 kb) Additional file 6:
**A phylogenetic tree of the individuals were constructed according to their genetic distances.** This figure shows the genetic relationships between studied animals based on their genomic kinships. (PDF 234 kb) Additional file 7:
**The Manhattan plot of GWAS for the femur length in the F**_**2**_ **population.** This figure shows the locus on SSC4 has been detected after correcting the effects of SSC7 and SSCX loci in the F_2_ population. (PDF 67 kb) Additional file 8:
**The Manhattan plot of the GWAS meta analysis.** This figure compares the Manhattan plot of the meta analysis with the F_2_ population to without it. (PDF 163 kb) Additional file 9:
**The chromosomal regions associated with the limb bone lengths under the assumption of the linkage phases being same in the four populations.** This table lists the loci identified under the assumption of the linkage phases being same in the four populations. (PDF 58 kb) Additional file 10:
**The chromosomal regions with** ***P*** **values ≤ 0.001 after genomic control.** This table lists the loci identified at 0.001 significant level after genomic control. (PDF 224 kb) Additional file 11:
**The linkage disequilibrium block in the significant genomic region on SSC7.** These figures show the LD blocks on SSC7 in the four populations and a 924-kb overlapping interval across those populations. (PDF 441 kb) Additional file 12:
**The tight linkage of the unassigned significant SNP with the mapped SNP.** This table lists the significant unmapped SNPs and their tightly linked mapped SNPs. (PDF 109 kb)

## Abbreviations

DNA: Deoxyribonucleic Acid
GWAS: Genome-wide Association Study
HMGA1: High Mobility Group AT-hook 1
kb: Kilo Base Pairs
LD: Linkage Disequilibrium
Mb: Mega Base Pairs
PLAG1: Pleiomorphic Adenoma Gene 1
QTL: Quantitative Trait Locus
SNP: Single Nucleotide Polymorphism
SSC: Sus Scrofa Chromosome
