# Supplementary material for: A meta analysis of genome-wide association studies for limb bone lengths in four pig populations
Source: BMC Genet. 2015 Jul 29;16:95. doi: 10.1186/s12863-015-0257-1 (PMC4518597; doi:10.1186/s12863-015-0257-1)
Supplement: Additional file 1: — The phenotypic correlation coefficients between the lengths of limb bones. This table provides the phenotypic correlation coefficients between the limb bone lengths in the four tested pig populations. (PDF 61 kb) [file 12863_2015_257_MOESM1_ESM.pdf]

Additional File 1 The phenotypic correlation coefficients between the lengths of limb bones

	Humerus	Ulna	Femur	Tibia
Erhualian				
Scapula	0.877	0.874	0.788	0.872
Humerus		0.884	0.825	0.905
Ulna			0.832	0.939
Femur				0.846
F_2				
Scapula	0.831	0.858	0.887	0.838
Humerus		0.899	0.886	0.814
Ulna			0.906	0.869
Femur				0.888
Laiwu				
Scapula	0.688	0.771	0.826	0.813
Humerus		0.801	0.799	0.763
Ulna			0.859	0.865
Femur				0.908
Sutai				
Scapula	0.873	0.854	0.924	0.882
Humerus		0.870	0.895	0.888
Ulna			0.901	0.912
Femur				0.941
ALL				
Scapula	0.845	0.875	0.870	0.832
Humerus		0.897	0.887	0.854
Ulna			0.894	0.889
Femur				0.901

All of the phenotypic correlation coefficients are significant with $P < 0.0001$.
