# Supplementary material for: A meta analysis of genome-wide association studies for limb bone lengths in four pig populations
Source: BMC Genet. 2015 Jul 29;16:95. doi: 10.1186/s12863-015-0257-1 (PMC4518597; doi:10.1186/s12863-015-0257-1)
Supplement: Additional file 2: — The distribution of the statistic (Z value) using in the GWAS meta analysis. This figure shows the distribution of Z value using in the meta-analysis. All of the statistics approximately follow the standard normal distribution. (PDF 144 kb) [file 12863_2015_257_MOESM2_ESM.pdf]

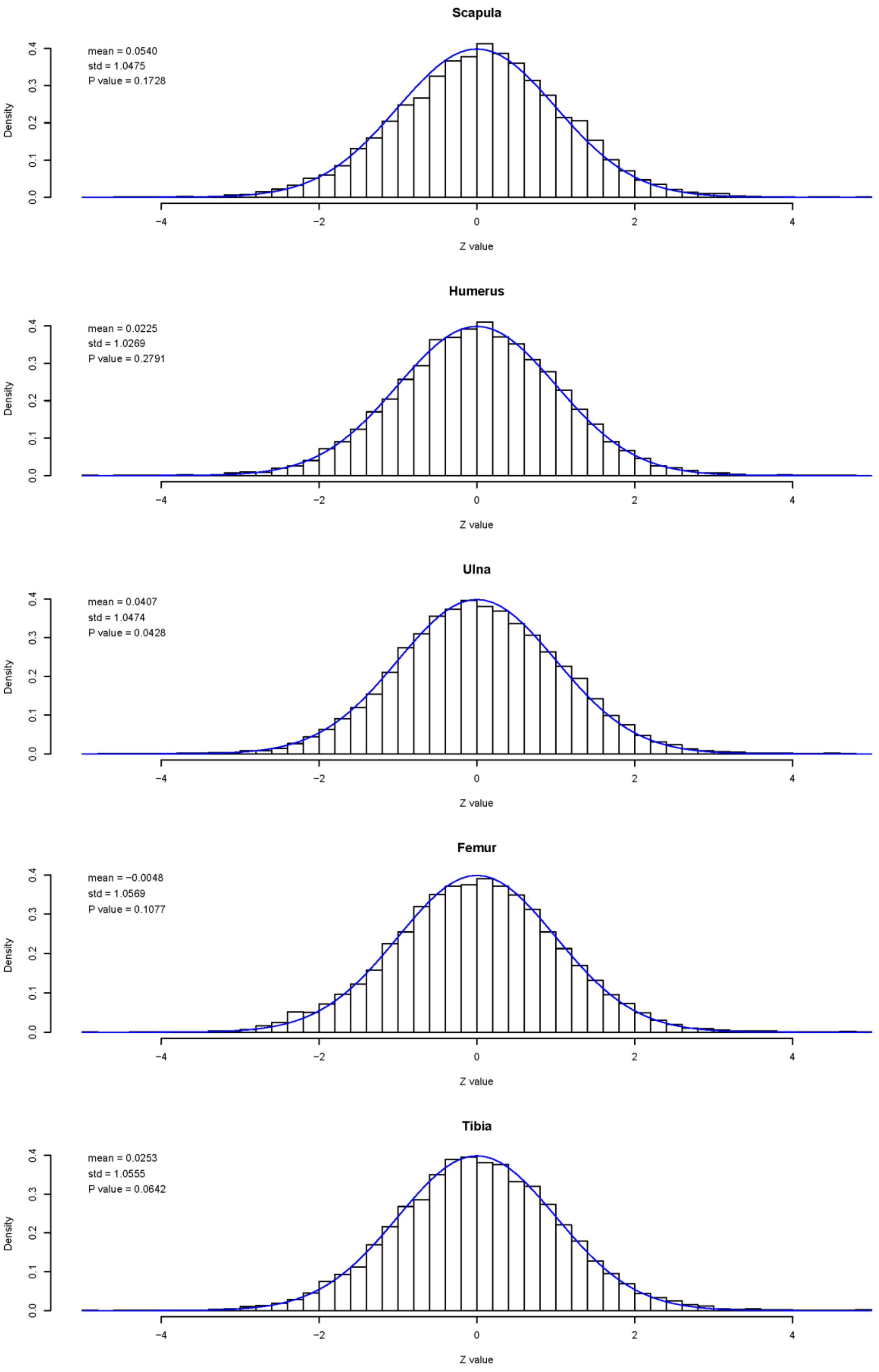

Additional File 2 The distribution of the statistic (*Z* value) using in the GWAS meta analysis. The blue curve is the probability density of the standard normal distribution, and the *P* value is obtained from a Kolmogorov-Smirnov test.
