# Supplementary material for: A meta analysis of genome-wide association studies for limb bone lengths in four pig populations
Source: BMC Genet. 2015 Jul 29;16:95. doi: 10.1186/s12863-015-0257-1 (PMC4518597; doi:10.1186/s12863-015-0257-1)
Supplement: Additional file 3: — The numbers of animals and SNPs passed through the quality control. This table lists the SNP and animal numbers and the average physical distance interval of adjacent SNPs using in the GWAS analysis. (PDF 113 kb) [file 12863_2015_257_MOESM3_ESM.pdf]

Additional File 3 The numbers of animals and SNPs passed through the quality control

Population	Animals	Mapped SNPs	Unmapped SNPs	Adjacent SNPs' interval, kb		
				Minimum	Average	Maximum
F_2	925	34636	4825	0	74.7	33542.4
Erhualian	331	24602	3492	0	105.1	37786.4
Laiwu	314	32058	4527	0	80.7	37786.4
Sutai	434	36341	4979	0	71.2	3755.8
Meta	2004	13595	1834	0	189.9	38806.1
