# Supplementary material for: A meta analysis of genome-wide association studies for limb bone lengths in four pig populations
Source: BMC Genet. 2015 Jul 29;16:95. doi: 10.1186/s12863-015-0257-1 (PMC4518597; doi:10.1186/s12863-015-0257-1)
Supplement: Additional file 4: — The schematic diagram of the first two principle components based on the genomic kinships. This figure presents the population stratification based on the genomic kinships in the four populations. (PDF 65 kb) [file 12863_2015_257_MOESM4_ESM.pdf]

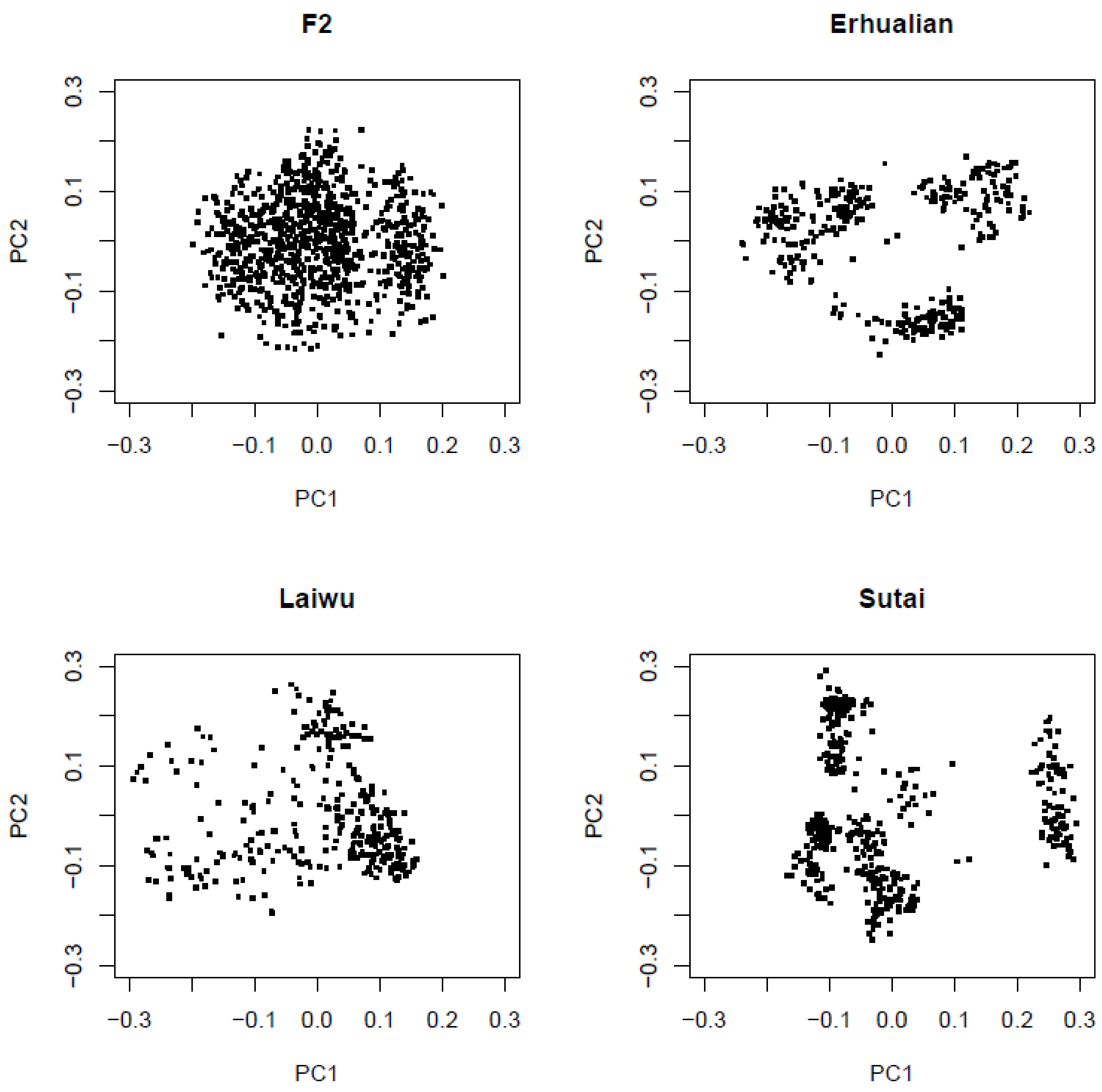

Additional File 4 The schematic diagram of the first two principle components based on the genomic kinships.
