# Supplementary material for: A meta analysis of genome-wide association studies for limb bone lengths in four pig populations
Source: BMC Genet. 2015 Jul 29;16:95. doi: 10.1186/s12863-015-0257-1 (PMC4518597; doi:10.1186/s12863-015-0257-1)
Supplement: Additional file 5: — The quantile-quantile plot for the limb bone lengths in the four populations. This figure indicates the residual inflation has been completely corrected by genomic control in GWAS. (PDF 265 kb) [file 12863_2015_257_MOESM5_ESM.pdf]

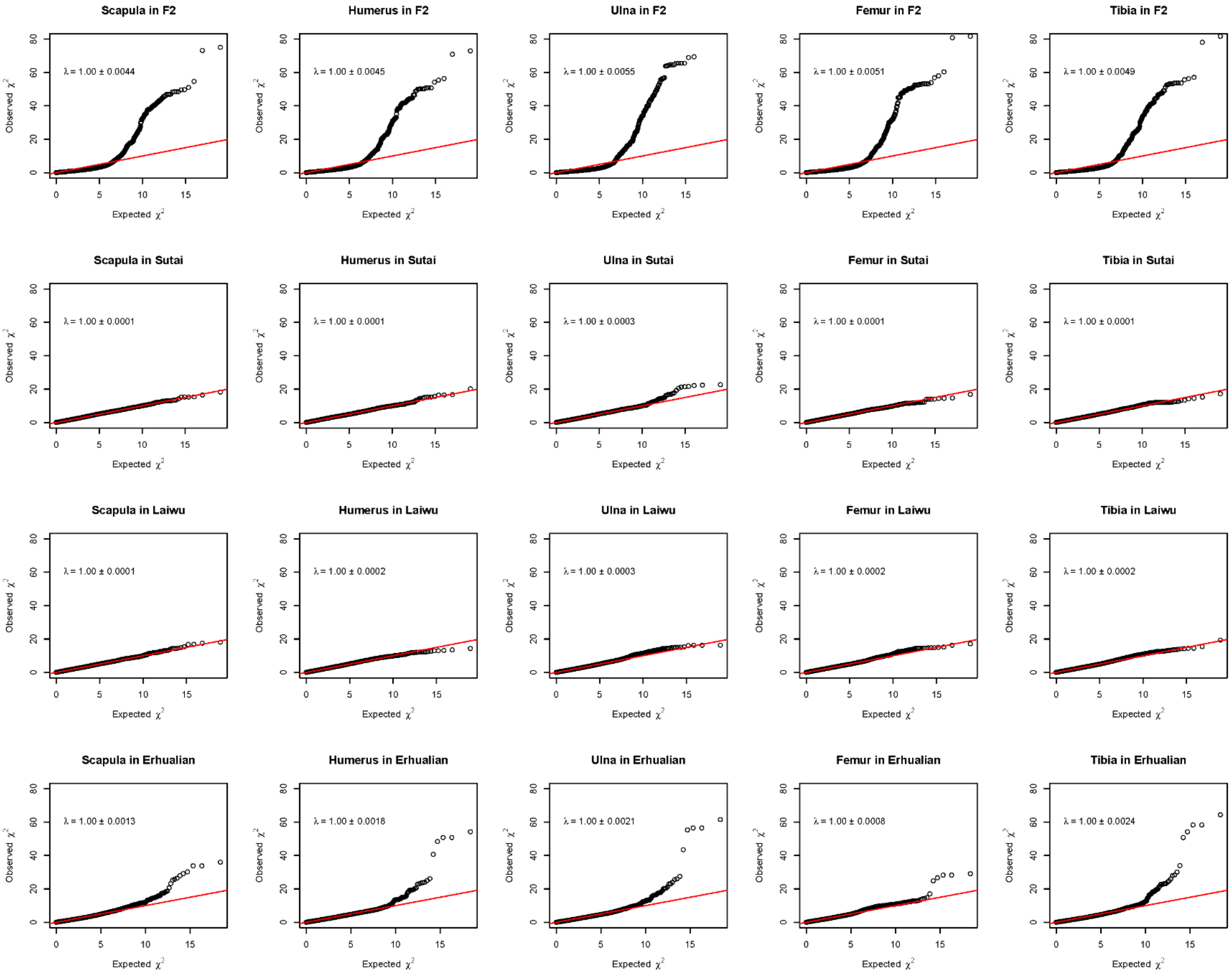

Additional File 5 The quantile-quantile (QQ) plot for the limb bone lengths in the four populations. X and y axes are the expected and observed values of association test statistics (chi-square), respectively. The estimated lambda (λ) and its standard error is showing in the figure.
