# Supplementary material for: A meta analysis of genome-wide association studies for limb bone lengths in four pig populations
Source: BMC Genet. 2015 Jul 29;16:95. doi: 10.1186/s12863-015-0257-1 (PMC4518597; doi:10.1186/s12863-015-0257-1)
Supplement: Additional file 6: — A phylogenetic tree of the individuals were constructed according to their genetic distances. This figure shows the genetic relationships between studied animals based on their genomic kinships. (PDF 234 kb) [file 12863_2015_257_MOESM6_ESM.pdf]

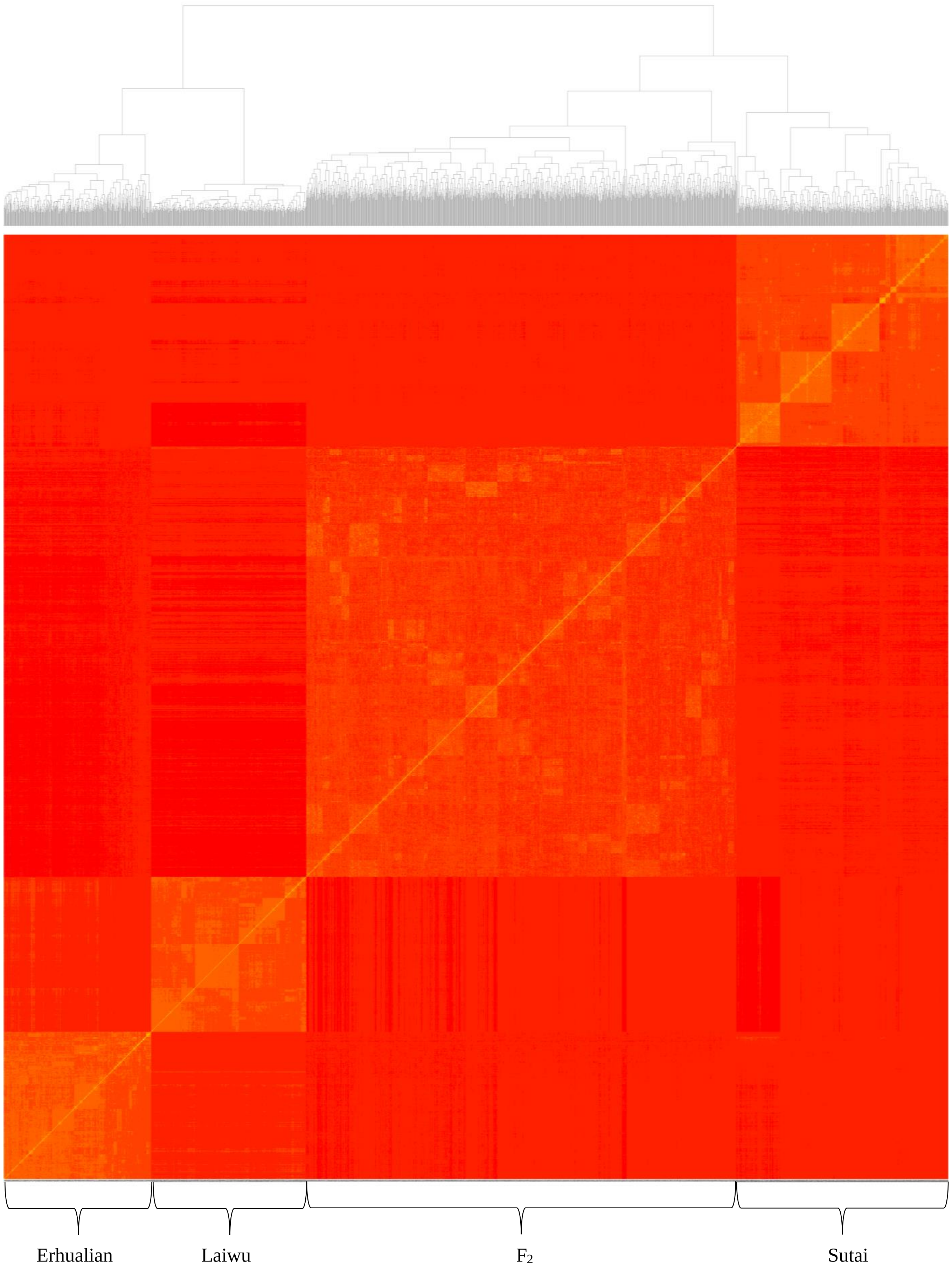

Additional File 6 A phylogenetic tree of the individuals were constructed according to their genetic distances. The clustering results completely agreed with the populations without exception.
