# Supplementary material for: A meta analysis of genome-wide association studies for limb bone lengths in four pig populations
Source: BMC Genet. 2015 Jul 29;16:95. doi: 10.1186/s12863-015-0257-1 (PMC4518597; doi:10.1186/s12863-015-0257-1)
Supplement: Additional file 7: — The Manhattan plot of GWAS for the femur length in the F 2 population. This figure shows the locus on SSC4 has been detected after correcting the effects of SSC7 and SSCX loci in the F2 population. (PDF 67 kb) [file 12863_2015_257_MOESM7_ESM.pdf]

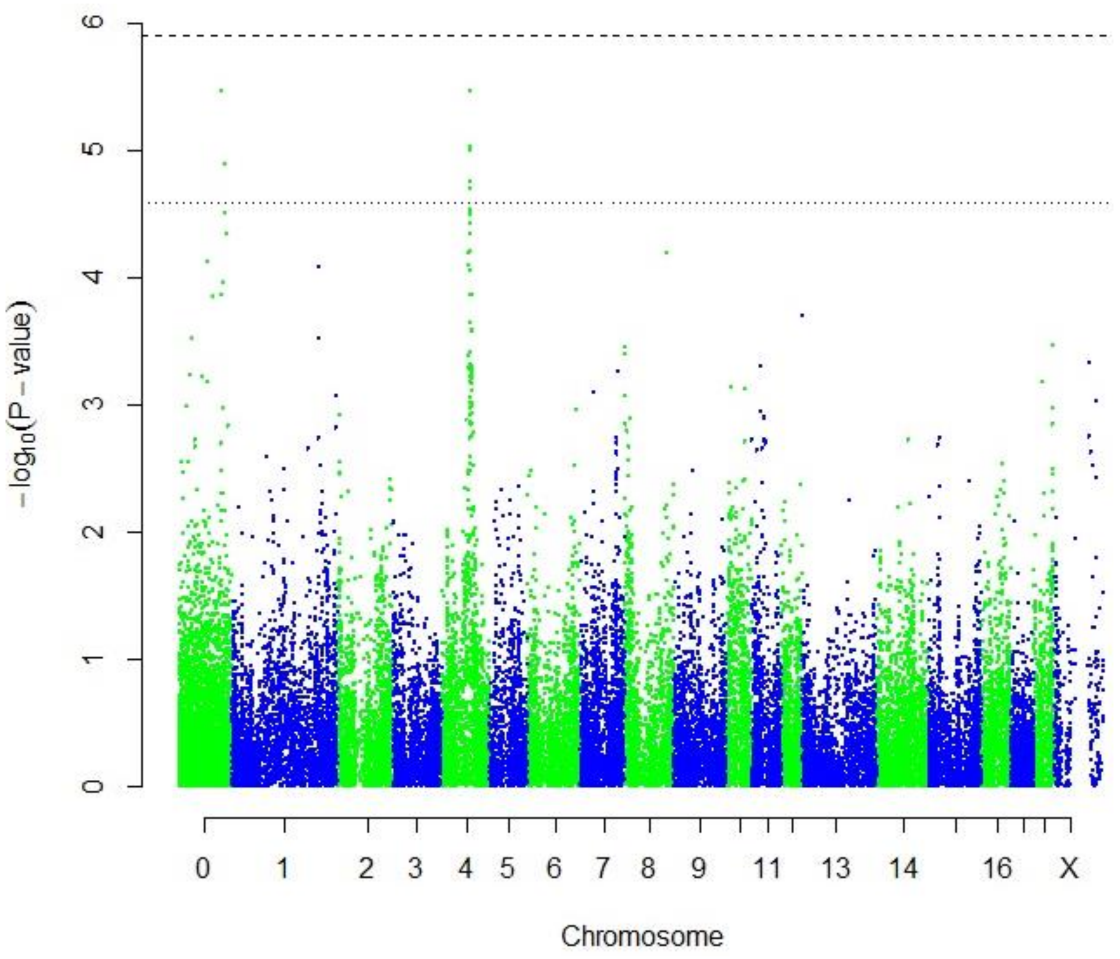

Additional File 7 The Manhattan plot of GWAS for the femur length in the F_2 population. The Manhattan plot showed the GWAS results after correcting for the effects of SSC7 and SSCX loci. The dashed and dotted horizontal lines indicate the 5% genome-wide and suggestive significant threshold values, respectively.
