# Supplementary material for: A meta analysis of genome-wide association studies for limb bone lengths in four pig populations
Source: BMC Genet. 2015 Jul 29;16:95. doi: 10.1186/s12863-015-0257-1 (PMC4518597; doi:10.1186/s12863-015-0257-1)
Supplement: Additional file 8: — The Manhattan plot of the GWAS meta analysis. This figure compares the Manhattan plot of the meta analysis with the F2 population to without it. (PDF 163 kb) [file 12863_2015_257_MOESM8_ESM.pdf]

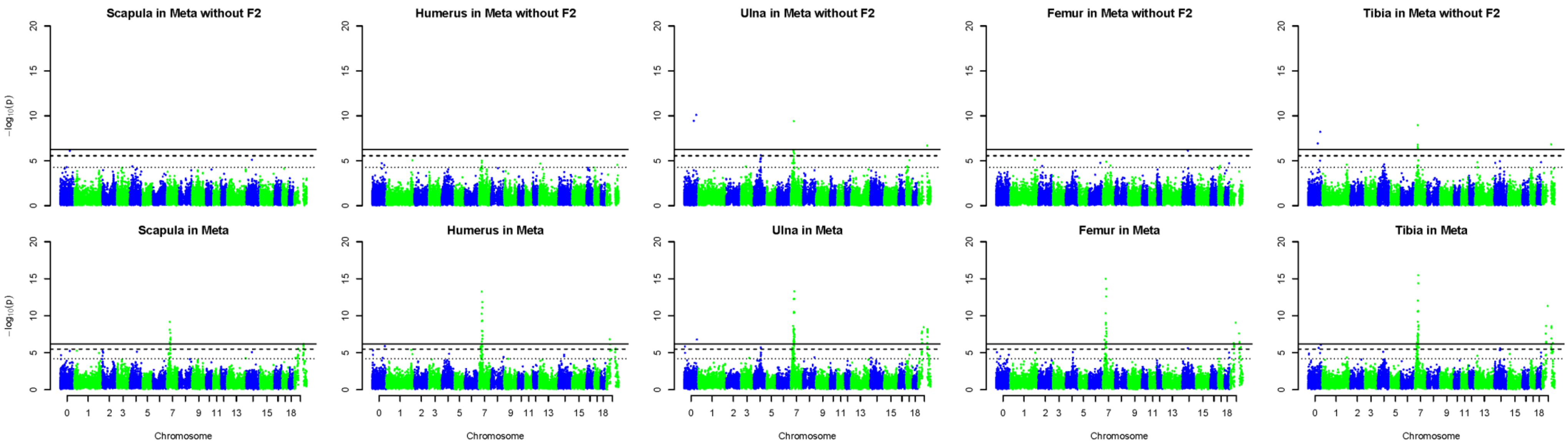

Additional File 8 The Manhattan plot of the GWAS meta analysis. Above. across the 3 pure breeds; Bottom. across the four populations. The dashed and dotted horizontal lines indicate the 5% genome-wide and suggestive significant threshold values, respectively.
