# Supplementary material for: A meta analysis of genome-wide association studies for limb bone lengths in four pig populations
Source: BMC Genet. 2015 Jul 29;16:95. doi: 10.1186/s12863-015-0257-1 (PMC4518597; doi:10.1186/s12863-015-0257-1)
Supplement: Additional file 9: — The chromosomal regions associated with the limb bone lengths under the assumption of the linkage phases being same in the four populations. This table lists the loci identified under the assumption of the linkage phases being same in the four populations. (PDF 58 kb) [file 12863_2015_257_MOESM9_ESM.pdf]

Additional File 9 The chromosomal regions associated with the limb bone lengths under the assumption of the linkage phases being same in the four populations

Trait	Chr[1]	Top SNP	Position, Mb	Effect ± SE	*P* Value[2]	N_{SNP}[3]
Scapula	1	ss131149408	282.04	-0.164±0.039	2.43×10^{05}	1
Scapula	2	ss131211507	3.61	-0.139±0.033	2.70×10^{05}	1
Ulna	4	ss131269439	81.71	0.195±0.041	$1.93\times10^{06*}$	1
Femur	4	ss131270306	84.98	0.163±0.040	4.72×10^{05}	1
Humerus	4	ss131270306	84.98	0.164±0.038	1.41×10^{05}	1
Femur	7	ss131342596	33.18	-0.232±0.037	$5.46\times10^{10**}$	3
Scapula	7	ss131342596	33.18	-0.194±0.040	$9.93\times10^{07*}$	1
Humerus	7	ss131347175	40.85	-0.190±0.034	$2.40\times10^{08**}$	4
Tibia	7	ss131347175	40.85	-0.224±0.034	$7.51\times10^{11**}$	6
Ulna	7	ss131347175	40.85	-0.233±0.038	$6.90\times10^{10**}$	8
Humerus	8	ss131065181	145.99	-0.131±0.032	3.86×10^{05}	1
Ulna	17	ss131546319	37.50	0.180±0.045	6.46×10^{05}	1
Tibia	18	ss107862415	52.79	0.143±0.035	3.39×10^{05}	1
Humerus	X	ss478943984	45.12	0.083±0.019	7.59×10^{06}	4
Femur	X	ss23131102	63.65	-0.133±0.022	$8.67\times10^{10**}$	3
Tibia	X	ss23131102	63.65	-0.140±0.020	$4.67\times10^{12**}$	2
Ulna	X	ss23131102	63.65	-0.129±0.022	$3.55\times10^{09**}$	2
Scapula	X	ss131070541	106.48	0.123±0.026	$2.72\times10^{06*}$	2

Notes: 1. Chromosome; 2. The *P* value was corrected by genomic control. **: 1% genome-wide significant; *: 5% genome-wide significant; without *: suggestive significant; 3. number of SNPs that surpass the suggestive significance level.
