# Supplementary material for: A meta analysis of genome-wide association studies for limb bone lengths in four pig populations
Source: BMC Genet. 2015 Jul 29;16:95. doi: 10.1186/s12863-015-0257-1 (PMC4518597; doi:10.1186/s12863-015-0257-1)
Supplement: Additional file 10: — The chromosomal regions with P values ≤ 0.001 after genomic control. This table lists the loci identified at 0.001 significant level after genomic control. (PDF 224 kb) [file 12863_2015_257_MOESM10_ESM.pdf]

Additional File 10 The chromosomal regions with P values ≤ 0.001 after genomic control

Chr[1]	Trait	Population	Top SNP	Position, Mb	Effect ± S.E.	P Value[2]	N_{SNP}[3]
1	Humerus	Laiwu	ss120022060	33.49	0.294±0.077	2.45×10^{-4}	6
1	Ulna	Meta	ss131133913	176.13	0.129±0.035	2.30×10^{-4}	8
1	Ulna	Sutai	ss107801243	248.80	0.263±0.072	4.48×10^{-4}	4
1	Ulna	Laiwu	ss107826847	280.64	0.256±0.069	6.64×10^{-4}	1
1	Humerus	Sutai	ss131155630	297.46	0.162±0.046	5.89×10^{-4}	2
1	Femur	Laiwu	ss107831074	303.97	0.229±0.058	3.24×10^{-4}	4
1	Scapula	Erhualian	ss131038377	309.79	0.461±0.125	6.53×10^{-4}	1
2	Femur	Sutai	ss107824650	28.25	0.217±0.056	1.94×10^{-4}	2
2	Humerus	Meta	ss131103405	44.81	0.117±0.031	1.56×10^{-4}	4
2	Femur	Laiwu	ss107845729	46.27	0.472±0.130	9.07×10^{-4}	2
2	Humerus	Sutai	ss131189210	50.64	0.175±0.052	9.39×10^{-4}	1
2	Tibia	Meta	ss131564921	127.15	0.136±0.035	8.72×10^{-5}	4
2	Scapula	Laiwu	ss131201108	128.42	0.404±0.110	3.16×10^{-4}	4
2	Femur	Meta	ss131208474	154.33	0.155±0.040	1.11×10^{-4}	7
3	Femur	Meta	ss131231647	5.61	0.118±0.032	2.03×10^{-4}	3
3	Femur	Sutai	ss131215509	9.48	0.291±0.083	7.30×10^{-4}	1
3	Tibia	Meta	ss131222267	13.56	0.146±0.040	2.94×10^{-4}	6
3	Tibia	Sutai	ss131222267	13.56	0.354±0.082	3.41×10^{-5}	3
3	Humerus	Meta	ss107841158	32.73	0.150±0.038	7.93×10^{-5}	9
3	Scapula	Meta	ss131217224	60.95	0.144±0.037	8.79×10^{-5}	16
3	Scapula	Laiwu	ss131217606	62.56	0.274±0.070	1.16×10^{-4}	16
4	Femur	Sutai	ss131249064	14.59	0.479±0.137	8.05×10^{-4}	1
4	Tibia	Erhualian	ss478940911	65.72	0.477±0.105	3.67×10^{-5}	2
4	Femur	Laiwu	ss478944380	77.03	0.332±0.073	3.56×10^{-5}	39
4	Tibia	Laiwu	ss478944380	77.03	0.297±0.068	8.61×10^{-5}	37
4	Humerus	Laiwu	ss131267615	77.19	0.303±0.086	6.52×10^{-4}	2
4	Ulna	Laiwu	ss478940989	77.58	0.355±0.081	5.51×10^{-5}	59
4	Scapula	Sutai	ss131245760	132.73	0.265±0.068	1.70×10^{-4}	3

5	Femur	Laiwu	ss131288413	6.80	0.427±0.111	4.18×10^{-4}	3
5	Tibia	Laiwu	ss131288413	6.80	0.371±0.099	7.22×10^{-4}	1
5	Humerus	Laiwu	ss131288413	6.80	0.414±0.118	7.55×10^{-4}	1
5	Scapula	Laiwu	ss131288413	6.80	0.388±0.111	6.05×10^{-4}	1
5	Tibia	Meta	ss131294686	87.76	0.138±0.040	5.35×10^{-4}	2
6	Tibia	Meta	ss131308626	13.67	0.129±0.034	1.41×10^{-4}	2
6	Scapula	Erhualian	ss131566460	126.22	0.505±0.121	1.26×10^{-4}	6
6	Scapula	Meta	ss120025426	131.83	0.132±0.034	1.33×10^{-4}	3
6	Femur	Meta	ss107911098	135.14	0.137±0.036	1.22×10^{-4}	1
6	Ulna	Meta	ss131045176	136.99	0.118±0.036	9.11×10^{-4}	1
6	Ulna	Erhualian	ss131117950	151.05	0.224±0.056	2.70×10^{-4}	1
7	Ulna	Sutai	ss478941558	9.77	0.271±0.074	3.99×10^{-4}	2
7	Ulna	Laiwu	ss131356028	59.36	0.475±0.130	7.40×10^{-4}	3
7	Scapula	Sutai	ss107839060	91.17	0.249±0.069	5.03×10^{-4}	2
7	Femur	Laiwu	ss107823551	94.81	0.454±0.123	7.83×10^{-4}	1
8	Ulna	Erhualian	ss131567089	20.12	0.222±0.057	4.40×10^{-4}	1
8	Femur	Meta	ss120029889	23.52	0.112±0.032	4.88×10^{-4}	1
8	Humerus	Laiwu	ss478934975	82.40	0.327±0.090	4.96×10^{-4}	3
8	Femur	Erhualian	ss131056090	110.82	0.573±0.149	3.70×10^{-4}	8
8	Tibia	Meta	ss131375845	114.38	0.108±0.031	4.08×10^{-4}	3
8	Tibia	Erhualian	ss131048831	122.66	0.341±0.090	5.45×10^{-4}	2
8	Scapula	Meta	ss131377162	127.82	0.154±0.039	7.72×10^{-5}	3
8	Scapula	Erhualian	ss131567419	129.88	0.273±0.074	6.11×10^{-4}	3
8	Scapula	Sutai	ss131380315	141.98	0.249±0.059	5.01×10^{-5}	5
8	Ulna	Sutai	ss107801256	142.28	0.239±0.067	5.70×10^{-4}	1
9	Humerus	Erhualian	ss131414482	17.34	0.246±0.059	8.87×10^{-5}	1
9	Femur	Meta	ss131390657	47.38	0.163±0.044	1.85×10^{-4}	2
9	Humerus	Meta	ss131390657	47.38	0.160±0.040	6.54×10^{-5}	1
9	Scapula	Meta	ss131395613	64.68	0.163±0.042	9.41×10^{-5}	16

9	Scapula	Sutai	ss107811992	65.15	0.236 ± 0.058	9.61×10^{-5}	16
9	Humerus	Laiwu	ss131036120	118.55	0.374 ± 0.095	1.57×10^{-4}	2
9	Ulna	Meta	ss107824562	143.33	0.129 ± 0.039	8.47×10^{-4}	2
10	Ulna	Meta	ss131432739	8.58	0.118 ± 0.032	2.54×10^{-4}	1
10	Ulna	Sutai	ss107845583	11.61	0.211 ± 0.060	7.55×10^{-4}	1
10	Femur	Sutai	ss107845583	11.61	0.207 ± 0.058	5.87×10^{-4}	1
10	Femur	Meta	ss107845583	11.61	0.111 ± 0.033	8.01×10^{-4}	1
10	Scapula	Sutai	ss107799199	12.05	0.194 ± 0.053	3.41×10^{-4}	1
10	Humerus	Meta	ss107816645	56.40	0.155 ± 0.046	7.28×10^{-4}	1
10	Scapula	Meta	ss131428094	63.71	0.133 ± 0.036	2.02×10^{-4}	1
10	Tibia	Meta	ss131119260	67.88	0.123 ± 0.032	1.14×10^{-4}	1
10	Scapula	Laiwu	ss120031824	68.20	0.404 ± 0.114	5.08×10^{-4}	2
10	Scapula	Erhualian	ss478939215	72.26	0.288 ± 0.079	8.12×10^{-4}	1
11	Femur	Meta	ss131451929	80.76	0.108 ± 0.032	9.05×10^{-4}	1
11	Femur	Laiwu	ss131451965	80.80	0.246 ± 0.066	6.36×10^{-4}	1
11	Scapula	Laiwu	ss131451965	80.80	0.288 ± 0.069	4.17×10^{-5}	3
11	Humerus	Laiwu	ss131452227	81.16	0.290 ± 0.084	8.43×10^{-4}	1
11	Tibia	Meta	ss131453457	83.47	0.106 ± 0.030	4.87×10^{-4}	2
12	Femur	Meta	ss131475589	2.52	0.118 ± 0.030	1.13×10^{-4}	8
12	Tibia	Meta	ss131475589	2.52	0.098 ± 0.028	5.96×10^{-4}	1
12	Scapula	Meta	ss131476871	2.88	0.106 ± 0.031	7.93×10^{-4}	1
12	Femur	Sutai	ss131568357	2.98	0.177 ± 0.050	6.65×10^{-4}	1
12	Tibia	Sutai	ss131568357	2.98	0.177 ± 0.051	9.00×10^{-4}	1
12	Tibia	Laiwu	ss131568432	25.58	0.352 ± 0.086	2.28×10^{-4}	2
12	Ulna	Meta	ss478937246	32.01	0.133 ± 0.035	1.29×10^{-4}	3
12	Scapula	Sutai	ss131063005	34	0.240 ± 0.066	4.39×10^{-4}	3
12	Humerus	Meta	ss131063005	34.00	0.102 ± 0.029	4.57×10^{-4}	3
12	Humerus	Sutai	ss131035905	42.38	0.201 ± 0.052	1.59×10^{-4}	3
12	Ulna	Laiwu	ss107812785	58.95	0.272 ± 0.066	1.41×10^{-4}	3

13	Humerus	Sutai	ss131480187	17.49	0.190±0.051	2.85×10^{-4}	2
13	Tibia	Laiwu	ss131488021	38.87	0.360±0.091	3.79×10^{-4}	1
13	Humerus	Meta	ss131488171	40.28	0.127±0.032	8.63×10^{-5}	8
13	Femur	Sutai	ss131488521	45.20	0.317±0.089	6.38×10^{-4}	3
13	Femur	Meta	ss107816469	59.17	0.133±0.038	4.63×10^{-4}	2
13	Ulna	Sutai	ss131477405	185.61	0.254±0.069	3.74×10^{-4}	1
13	Ulna	Erhualian	ss131477463	185.84	0.232±0.063	8.12×10^{-4}	1
13	Ulna	Meta	ss120025165	199.55	0.151±0.039	9.03×10^{-5}	4
13	Scapula	Laiwu	ss131480458	203.12	0.415±0.118	6.05×10^{-4}	2
13	Scapula	Erhualian	ss478937383	213.34	0.256±0.069	6.03×10^{-4}	2
14	Ulna	Sutai	ss131508262	18.18	0.264±0.069	2.34×10^{-4}	3
14	Humerus	Sutai	ss131511298	37.25	0.267±0.075	5.48×10^{-4}	2
14	Scapula	Erhualian	ss107864658	48.54	0.372±0.101	7.04×10^{-4}	3
14	Tibia	Sutai	ss120028609	56.27	0.278±0.077	4.99×10^{-4}	6
14	Femur	Sutai	ss131515687	64.87	0.239±0.060	1.27×10^{-4}	15
14	Ulna	Laiwu	ss107874852	152.52	0.271±0.073	6.05×10^{-4}	2
14	Humerus	Laiwu	ss107874852	152.52	0.267±0.074	5.02×10^{-4}	3
15	Scapula	Sutai	ss131522154	4.50	0.325±0.087	2.80×10^{-4}	4
15	Ulna	Meta	ss131525838	11.30	0.109±0.032	7.01×10^{-4}	1
15	Tibia	Sutai	ss131521797	19.49	0.245±0.071	9.11×10^{-4}	2
15	Humerus	Meta	ss107880152	21.18	0.172±0.049	4.71×10^{-4}	2
15	Femur	Meta	ss131081142	35.57	0.119±0.035	6.99×10^{-4}	3
15	Femur	Sutai	ss131523849	40.78	0.314±0.092	9.95×10^{-4}	2
15	Humerus	Sutai	ss478944261	45.84	0.367±0.087	4.43×10^{-5}	2
15	Ulna	Sutai	ss107859991	72.94	0.354±0.100	6.93×10^{-4}	1
15	Ulna	Laiwu	ss131529477	137.70	0.282±0.079	9.47×10^{-4}	1
15	Scapula	Meta	ss131569463	150.37	0.127±0.036	4.51×10^{-4}	4
16	Scapula	Laiwu	ss131539813	9.66	0.472±0.131	4.01×10^{-4}	1
16	Femur	Laiwu	ss131542184	17.47	0.384±0.098	3.40×10^{-4}	2

16	Humerus	Laiwu	ss131542184	17.47	0.393±0.105	3.26×10^{-4}	4
16	Ulna	Laiwu	ss131041188	20.97	0.395±0.106	5.65×10^{-4}	5
16	Femur	Meta	ss107846705	24.77	0.119±0.034	4.71×10^{-4}	4
16	Ulna	Meta	ss131535731	44.51	0.148±0.041	2.54×10^{-4}	5
16	Scapula	Meta	ss131540384	81.62	0.124±0.035	4.88×10^{-4}	3
16	Scapula	Sutai	ss131541031	82.84	0.204±0.059	8.74×10^{-4}	1
16	Humerus	Meta	ss131103922	84.38	0.121±0.030	6.97×10^{-5}	6
17	Humerus	Meta	ss131064820	3.751	0.129±0.033	9.01×10^{-5}	4
17	Humerus	Laiwu	ss131550383	6.35	0.264±0.073	5.05×10^{-4}	1
17	Tibia	Sutai	ss120019525	9.40	0.322±0.079	9.71×10^{-5}	9
17	Tibia	Meta	ss131542879	13.83	0.125±0.032	1.09×10^{-4}	8
17	Femur	Laiwu	ss131543061	16.49	0.295±0.076	4.21×10^{-4}	1
17	Tibia	Laiwu	ss131543061	16.49	0.267±0.070	5.16×10^{-4}	1
17	Humerus	Sutai	ss107853195	17.42	0.262±0.064	7.93×10^{-5}	2
17	Femur	Sutai	ss107823760	22.50	0.237±0.056	4.10×10^{-5}	10
17	Scapula	Meta	ss107823760	22.50	0.146±0.039	1.82×10^{-4}	2
18	Scapula	Meta	ss107867988	14.93	0.118±0.036	9.49×10^{-4}	1
18	Scapula	Laiwu	ss131555889	36.26	0.336±0.084	9.10×10^{-5}	4
18	Ulna	Meta	ss107835048	52.79	0.117±0.034	5.69×10^{-4}	2
18	Humerus	Meta	ss131558380	55.27	0.112±0.031	2.53×10^{-4}	1
X	Tibia	Sutai	ss131561906	56.72	0.135±0.037	5.29×10^{-4}	33
X	Tibia	Erhualian	ss478935724	102.94	0.186±0.041	3.82×10^{-5}	1

Notes: 1. Chromosome; 2. The *P* value was corrected by genomic control. **: 1% genome-wide significant; *: 5% genome-wide significant; without *: suggestive significant; 3. number of SNPs that surpass the suggestive significance level.
