# Supplementary material for: A meta analysis of genome-wide association studies for limb bone lengths in four pig populations
Source: BMC Genet. 2015 Jul 29;16:95. doi: 10.1186/s12863-015-0257-1 (PMC4518597; doi:10.1186/s12863-015-0257-1)
Supplement: Additional file 11: — The linkage disequilibrium block in the significant genomic region on SSC7. These figures show the LD blocks on SSC7 in the four populations and a 924-kb overlapping interval across those populations. (PDF 441 kb) [file 12863_2015_257_MOESM11_ESM.pdf]

a

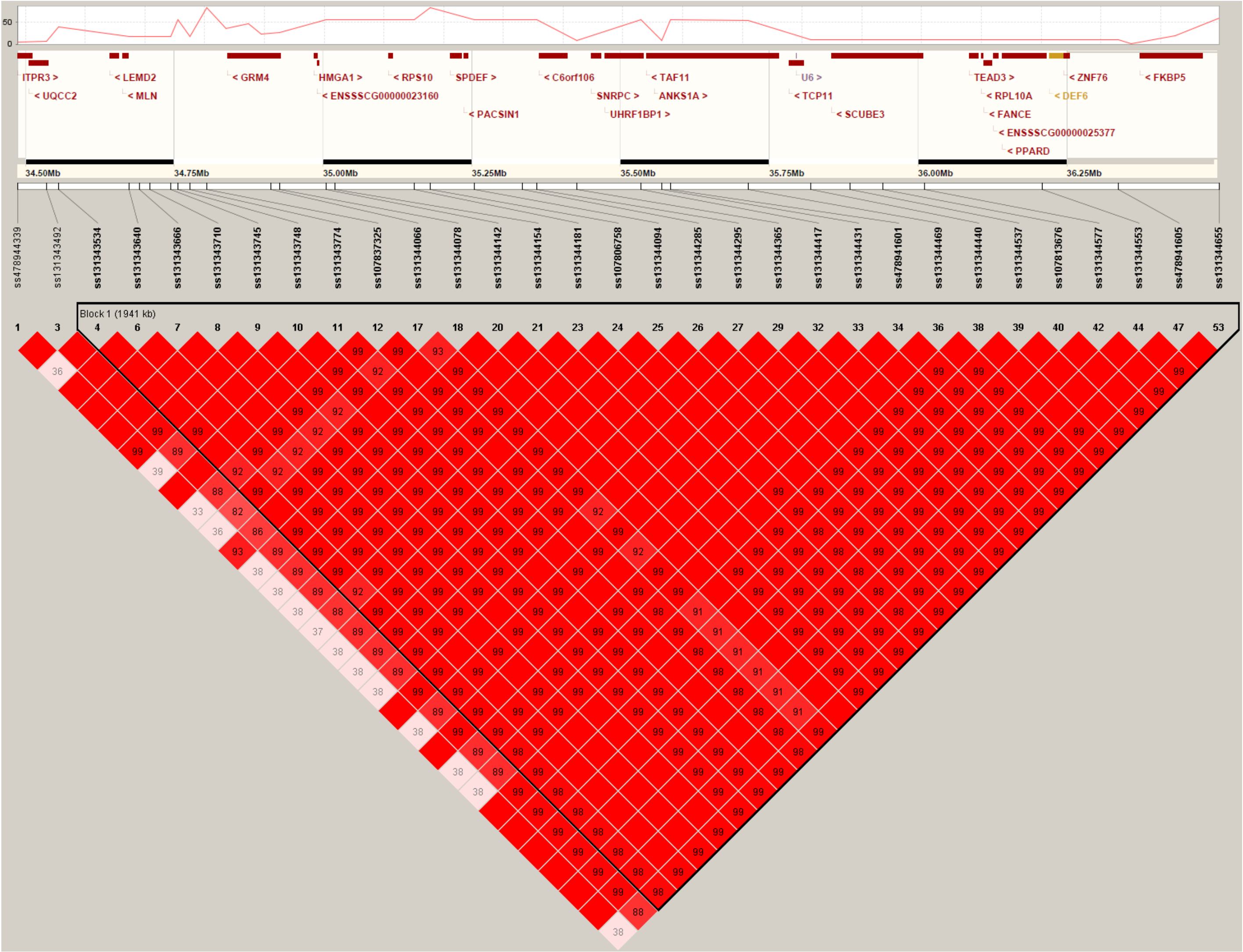

50
0
ITPR3 >
< UQCC2
< LEMD2
< MLN
< GRM4
HMGA1 >
< ENSSSCG00000023160
< RPS10
SPDEF >
< PACSIN1
< C6orf106
SNRPC >
UHRF1BP1 >
< TAF11
ANKS1A >
U6 >
< TCP11
< SCUBE3
TEAD3 >
< RPL10A
< FANCE
< ENSSSCG00000025377
< PPARD
< ZNF76
< DEF6
< FKBP5
34.50Mb
34.75Mb
35.00Mb
35.25Mb
35.50Mb
35.75Mb
36.00Mb
36.25Mb
ss478944339
ss131343492
ss131343534
ss131343640
ss131343666
ss131343710
ss131343745
ss131343748
ss131343774
ss107837325
ss131344066
ss131344078
ss131344142
ss131344154
ss131344181
ss107806758
ss131344094
ss131344285
ss131344295
ss131344365
ss131344417
ss131344431
ss478941601
ss131344469
ss131344440
ss131344537
ss107813676
ss131344577
ss131344553
ss478941605
ss131344655
Block 1 (1941 kb)
1 3 4 6 7 8 9 10 11 12 17 18 20 21 23 24 25 26 27 29 32 33 34 36 38 39 40 42 44 47 53

b

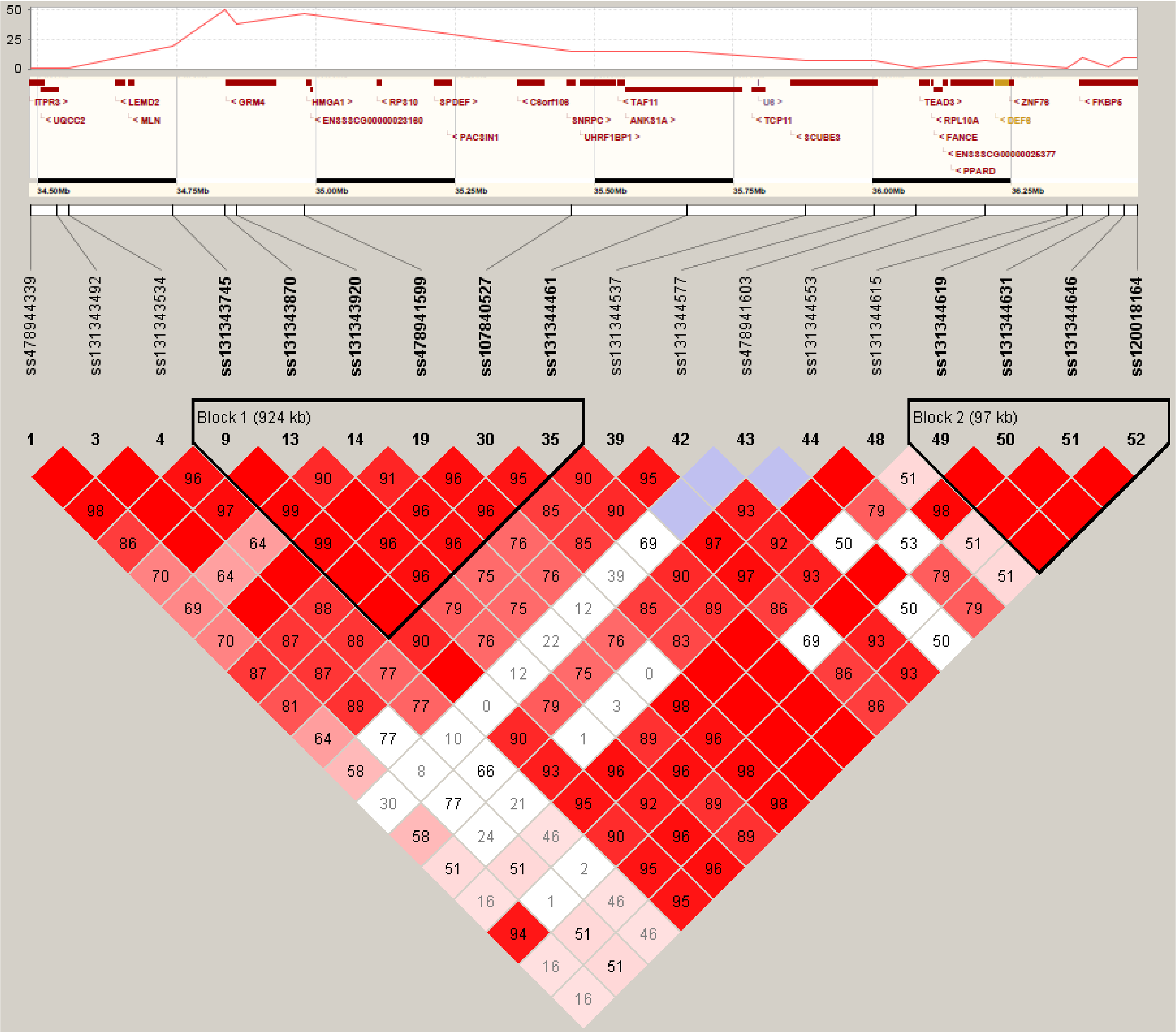
50
25
0
ITPR3
UQCC2
LEMD2
MLN
GRM4
HMGA1
ENSSSCG00000023160
RPS10
SPDEF
PACSIN1
C6orf106
SNRPC
UHRF1BP1
TAF11
ANKS1A
U6
TCP11
SCUBE3
TEAD3
RPL10A
FANCE
ENSSSCG00000025377
PPARD
ZNF76
DEF6
FKBP5
34.50Mb
34.75Mb
35.00Mb
35.25Mb
35.50Mb
35.75Mb
36.00Mb
36.25Mb
ss478944339
ss131343492
ss131343534
ss131343745
ss131343870
ss131343920
ss478941599
ss107840527
ss131344461
ss131344537
ss131344577
ss478941603
ss131344553
ss131344615
ss131344619
ss131344631
ss131344646
ss120018164
Block 1 (924 kb)
Block 2 (97 kb)
1
3
4
9
13
14
19
30
35
39
42
43
44
48
49
50
51
52

c

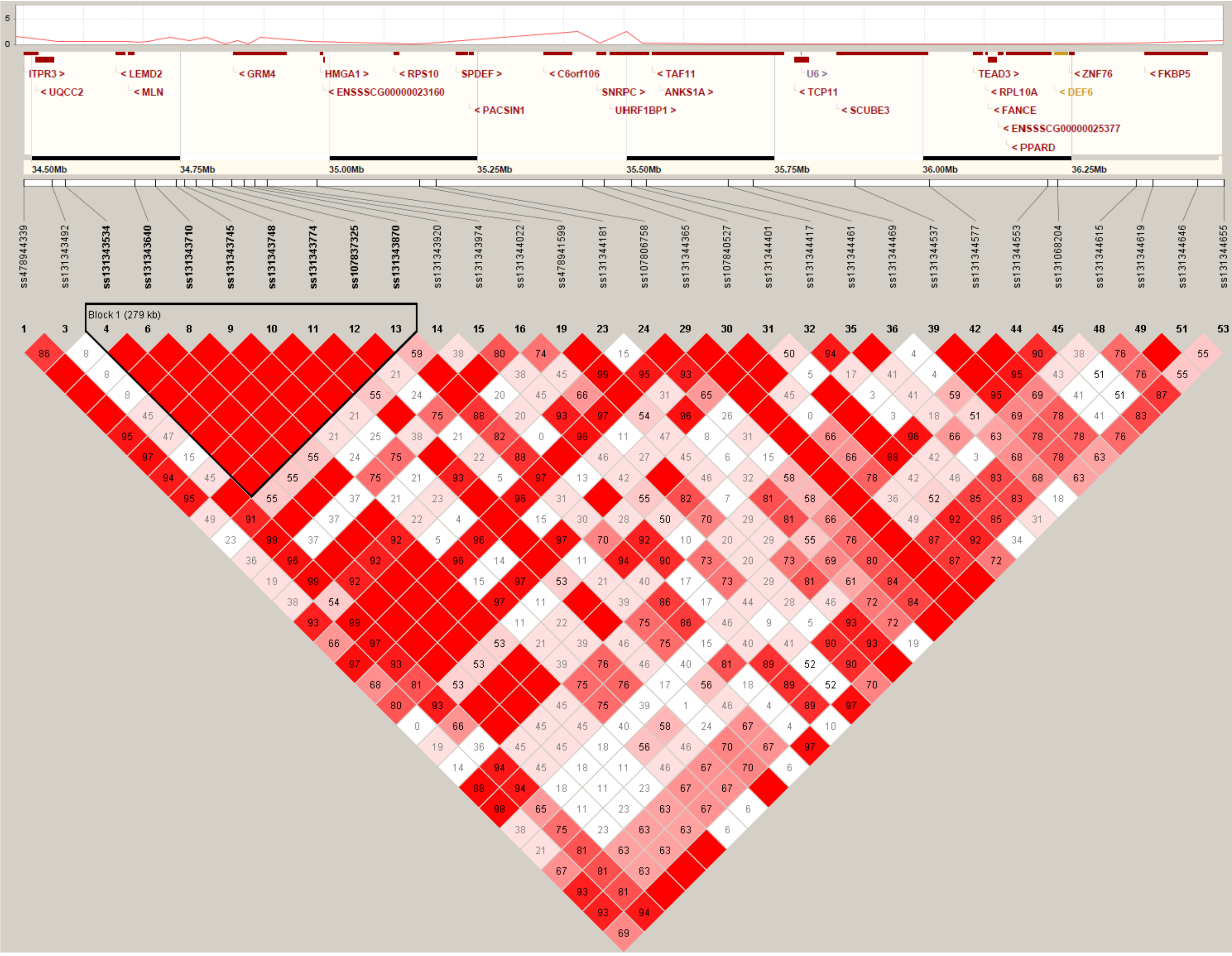

5
0
ITPR3 >
< UQCC2
< LEMD2
< MLN
< GRM4
HMGA1 >
< ENSSSCG00000023160
< RPS10
SPDEF >
< PACSIN1
< C6orf106
SNRPC >
UHRF1BP1 >
< TAF11
ANKS1A >
U6 >
< TCP11
< SCUBE3
TEAD3 >
< RPL10A
< FANCE
< ENSSSCG00000025377
< PPARD
< ZNF76
< DEF6
< FKBP5
34.50Mb
34.75Mb
35.00Mb
35.25Mb
35.50Mb
35.75Mb
36.00Mb
36.25Mb
ss478944339
ss131343492
ss131343534
ss131343640
ss131343710
ss131343745
ss131343748
ss131343774
ss107837325
ss131343870
ss131343920
ss131343974
ss131344022
ss478941599
ss131344181
ss107806758
ss131344365
ss107840527
ss131344401
ss131344417
ss131344461
ss131344469
ss131344537
ss131344577
ss131344553
ss131068204
ss131344615
ss131344619
ss131344646
ss131344655
Block 1 (279 kb)
1 3 4 6 8 9 10 11 12 13 14 15 16 19 23 24 29 30 31 32 35 36 39 42 44 45 48 49 51 53

d

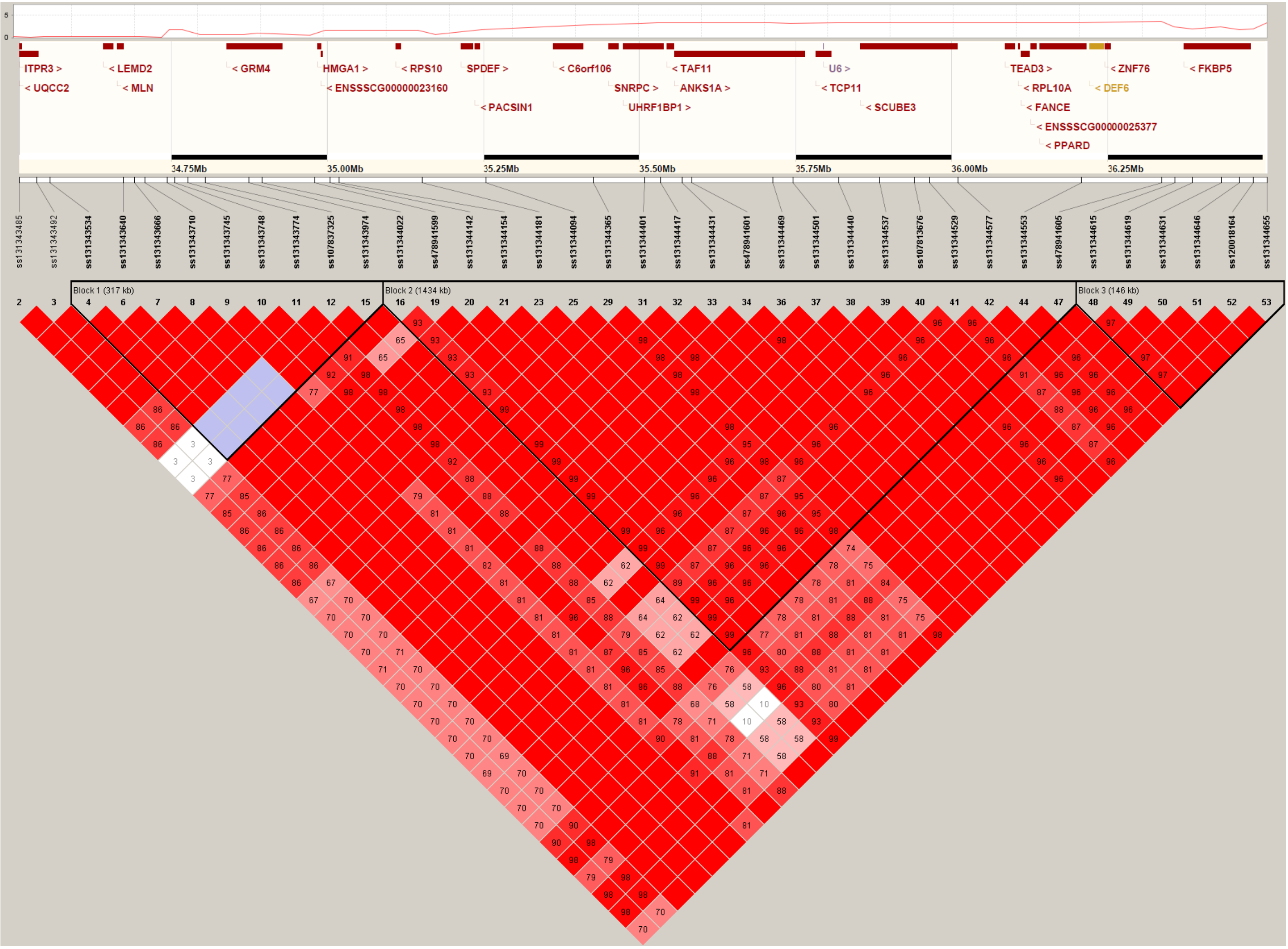

ITPR3 >
< UQCC2
< LEMD2
< MLN
< GRM4
HMGA1 >
< ENSSSCG00000023160
< RPS10
SPDEF >
< PACSIN1
< C6orf106
SNRPC >
UHRF1BP1 >
< TAF11
ANKS1A >
U6 >
< TCP11
< SCUBE3
TEAD3 >
< RPL10A
< FANCE
< ENSSSCG00000025377
< PPARD
< ZNF76
< DEF6
< FKBP5
34.75Mb
35.00Mb
35.25Mb
35.50Mb
35.75Mb
36.00Mb
36.25Mb
ss131343485
ss131343492
ss131343534
ss131343640
ss131343666
ss131343710
ss131343745
ss131343748
ss131343774
ss107837325
ss131343974
ss131344022
ss478941599
ss131344142
ss131344154
ss131344181
ss131344094
ss131344365
ss131344401
ss131344417
ss131344431
ss478941601
ss131344469
ss131344501
ss131344440
ss131344537
ss107813676
ss131344529
ss131344577
ss131344553
ss478941605
ss131344615
ss131344619
ss131344631
ss131344646
ss120018164
ss131344655
Block 1 (317 kb)
Block 2 (1434 kb)
Block 3 (146 kb)
2 3 4 6 7 8 9 10 11 12 15 16 19 20 21 23 25 29 31 32 33 34 36 37 38 39 40 41 42 44 47 48 49 50 51 52 53

e

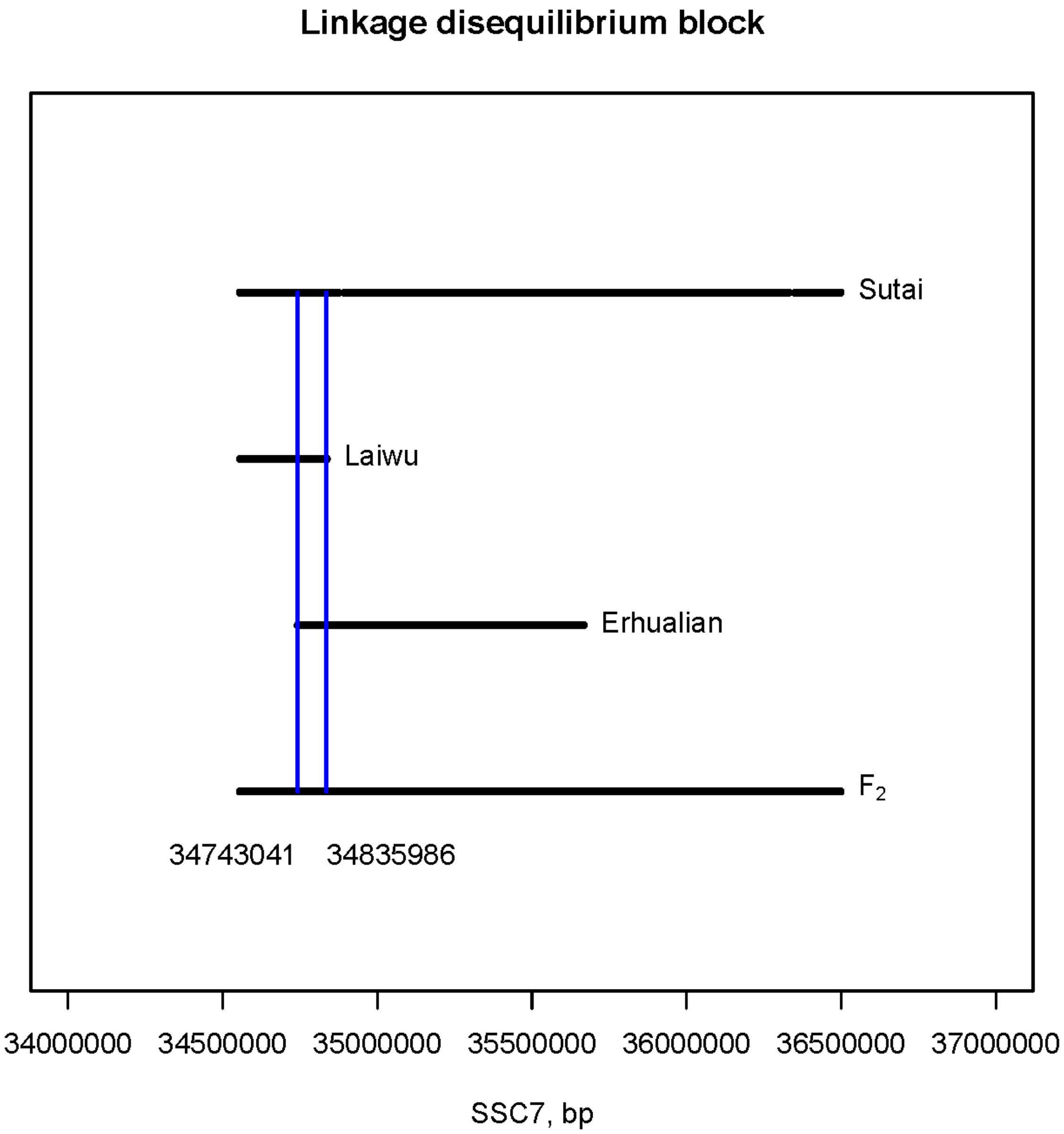

Additional File 11 The linkage disequilibrium (LD) block in the significant genomic region on SSC7. a. F_2; b. Erhualian; c. Laiwu; d. Sutai; e. LD block sharing among populations. Top: The curve of the negative $\log_{10}$ meta *P* value of the limb bone lengths; Middle: Annotated genes in the chromosomal region retrieved from the Ensemble genome browser; Bottom: LD block.
