# Supplementary material for: A meta analysis of genome-wide association studies for limb bone lengths in four pig populations
Source: BMC Genet. 2015 Jul 29;16:95. doi: 10.1186/s12863-015-0257-1 (PMC4518597; doi:10.1186/s12863-015-0257-1)
Supplement: Additional file 12: — The tight linkage of the unassigned significant SNP with the mapped SNP. This table lists the significant unmapped SNPs and their tightly linked mapped SNPs. (PDF 109 kb) [file 12863_2015_257_MOESM12_ESM.pdf]

Additional File 12 The tight linkage of the unassigned significant SNP with the mapped SNP

Unmapped SNP	Population	Associated trait	Tightly linked SNP			Recombination	LOD
			Name	Chromosome	Position, bp		
ss131338262	F_2	Scapula, Humerus, Tibia	ss131337985	7	21535371	0.0054	391.40
ss131338295	F_2	Scapula, Humerus, Tibia	ss131337985	7	21535371	0.0054	391.40
ss131345835	Meta	Humerus, Ulna, Tibia	ss131344469	7	35709335	0.0060	195.07
ss131348218	F_2	Scapula, Ulna, Femur, Tibia	ss131348350	7	43205140	0.0049	389.81
ss131101988	F_2	Scapula, Humerus, Ulna, Femur, Tibia	ss131342502	7	32997273	0.0016	404.24
ss131079954	F_2	Femur	ss131342502	7	32997273	0.0000	108.07
ss131158626	Meta	Femur	ss478937395	1	303886984	0.0000	119.21
ss131265188	Erhualian	Scapula	ss131265212	4	66284214	0.0024	301.78
ss131344757	F_2	Scapula, Humerus, Ulna, Femur, Tibia	ss131344469	7	35709335	0.0024	288.53
ss131347664	F_2	Scapula, Humerus, Ulna, Femur, Tibia	ss131347648	7	41624144	0.0021	323.89

ss478936101	F_2	Scapula, Humerus, Ulna, Femur, Tibia	ss131344469	7	35709335	0.0000	106.26
ss120019105	F_2	Scapula, Humerus, Ulna, Femur, Tibia	ss131344469	7	35709335	0.0000	113.49
ss478940923	Erhualian	Scapula	ss131265212	4	66284214	0.0000	136.07
ss478940923	Meta	Scapula	ss131265212	4	66284214	0.0000	136.07
ss131342382	F_2	Scapula, Humerus, Ulna, Femur, Tibia	ss131342502	7	32997273	0.0016	404.84
ss107902755	F_2	Scapula, Humerus, Tibia	ss107842725	7	24777963	0.0056	208.15
ss107879050	F_2	Scapula, Humerus, Ulna, Femur, Tibia	ss131342502	7	32997273	0.0016	404.24
ss107830715	Meta	Tibia	ss107865741	17	15101576	0.0000	95.73
ss107860263	Meta	Humerus, Ulna, Femur, Tibia	ss131344469	7	35709335	0.0022	213.89
ss107855265	F_2	Scapula, Humerus, Tibia	ss107842725	7	24777963	0.0056	208.15
